# C/EBP *α* is Essential for Gonadal but Not Inguinal White Adipose Tissue Formation in Mice

**DOI:** 10.1002/oby.70142

**Published:** 2026-02-11

**Authors:** Krista Y. Hu, Yu‐Lin Ma, Esme A. Dodge, Olivia A. B. Maguire, Caio V. Matias, Ryan P. Barney, Hector S. Himede, Juliana Gomez Pardo, Miriam Cepeda, Scott M. Gordon, Robert C. Bauer

**Affiliations:** ^1^ Cardiometabolic Genomics Program, Division of Cardiology, Department of Medicine Columbia University New York New York USA; ^2^ Department of Physiology University of Kentucky Lexington Kentucky USA; ^3^ Saha Cardiovascular Research Center University of Kentucky Lexington Kentucky USA

**Keywords:** *CEBPA*, GWAS, transcription factor, visceral adipose, WAT development

## Abstract

**Objective:**

The distribution of excess white adipose tissue (WAT) in obesity correlates with risk for comorbidities. Thus, understanding depot‐specific WAT developmental mechanisms is translationally relevant. SNPs near the gene *CEBPA* associate with waist to hip ratio, and while C/EBP*α* is a recognized regulator of adipogenesis, there is no previously known role for C/EBP*α* in regulating adipose distribution.

**Methods:**

We crossed *Cebpa* floxed mice to the AdipoQ‐Cre transgenic mouse strain, generating mice with adipocyte‐specific knockout of *Cebpa* (Cebpa_ASKO). Mice were phenotyped on a chow diet and after prolonged high‐fat diet (HFD) feeding.

**Results:**

Cebpa_ASKO mice almost entirely lack gonadal WAT (gWAT), while inguinal WAT (iWAT) is present in near normal amounts. Despite developing, Cebpa_ASKO iWAT contains fewer and larger adipocytes, fails to expand under HFD challenge, and is dysfunctional as evidenced by transcriptomics and functional studies. Finally, Cebpa_ASKO mice have lipid‐laden brown adipose tissue (BAT), increased hepatic triglycerides, and increased plasma cholesterol, all of which worsen with prolonged HFD feeding.

**Conclusions:**

These results highlight a previously unrecognized difference in the essentiality of C/EBP*α* for gWAT and iWAT development and highlight novel interorgan relationships between WAT and other metabolic tissues. Further studies of these specific mechanisms could have clinical relevance for targeting visceral adiposity in humans.

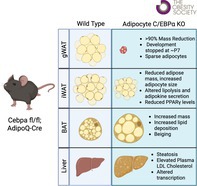

## Study Importance

- What is already known?
  ○ The transcription factor C/EBP*α* is a critical regulator of adipogenesis.
  ○ The genomic locus containing the gene *CEBPA* is associated with visceral adiposity in humans.
- What does this study add?
  ○ Adipocyte‐specific *Cebpa* knockout mice fail to develop gonadal white adipose tissue (WAT) due to stalled development in the perinatal period.
  ○ All other WAT depots develop normally but are dysfunctional, leading to numerous metabolic phenotypes in the knockout mice.
- How might these results change the direction of research?
  ○ Given the clinical relevance of visceral adipose, further elucidation of the depot‐specific mechanisms of C/EBP*α* may unveil novel avenues for therapeutic intervention.
  ○ The metabolic phenotypes present in our mice suggest novel relationships between WAT function and other metabolic organs worthy of further research.

## Graphical abstract

Human genetics has linked the gene *CEBPA* to waist to hip ratio and adipose distribution. We generated mice with AdipoQ‐Cre driven deletion of *Cebpa* and found these mice to have a specific reduction in gonadal white adipose tissue and myriad other metabolic phenotypes.

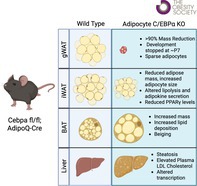

## 1 Introduction

Rates of obesity, defined as excess accumulation of white adipose tissue (WAT), continue to rise in the developed world. Obesity is well known to be associated with multiple metabolic comorbidities, including increased risk for cardiovascular disease (CVD), type 2 diabetes (T2D), and metabolic dysfunction‐associated steatotic liver disease, among others [1]. Interestingly, the distribution of this excess WAT can result in different risks for metabolic disease [2, 3]. There are two main types of WAT: subcutaneous white adipose tissue (scWAT) located under the skin and visceral white adipose tissue (vWAT) located inside the peritoneum in the abdominal area [4]. Humans with excess vWAT have nearly 10 times greater risk of poor metabolic outcomes, such as CVD and insulin resistance [2, 5, 6]. Conversely, excess accumulation of scWAT in humans is sometimes referred to as “metabolically healthy obesity,” as it is less associated with poor metabolic outcomes [2, 7]. Given these observations, identifying mechanisms that regulate the distribution of adipose tissue between scWAT and vWAT may elucidate factors that can be leveraged in the treatment of obesity and cardiometabolic disease.

Genome‐wide association studies (GWAS) have identified more than 1000 loci that associate with obesity traits in humans, including associations with body mass index (BMI), body fat percentage, waist to hip‐ratio (WHR), and WHR adjusted for BMI (WHRadjBMI), the last of which is a measure of visceral adiposity [8]. The 19q13 GWAS locus contains SNPs that are associated with numerous cardiometabolic traits including plasma triglycerides, plasma total cholesterol, T2D, BMI, and WHRadjBMI [9, 10]. There are multiple genes in the 19q13 GWAS locus, including CCAAT/enhancer binding protein alpha (*CEBPA*) (Figure S1) [9]. *CEBPA* encodes the protein C/EBP*α*, which is a transcription factor that, in coordination with C/EBPβ and peroxisome proliferator‐activated receptor gamma (PPAR*γ*), governs the adipocyte differentiation process. In particular, C/EBP*α* is known to work in a positive regulatory loop with PPAR*γ* to drive the adipogenic gene expression program [11, 12]. In mice, germline whole body *Cebpa* knockout is perinatal lethal, but restoration of hepatic *Cebpa* expression permits survival and the resulting mice are lipodystrophic and lack WAT [13]. While clearly required for adipogenesis, it is unclear how C/EBP*α* regulates adipose distribution and visceral adiposity specifically.

Given the GWAS association with WHRadjBMI, we hypothesized that adipocyte *Cebpa* plays a role in WAT distribution. To test this hypothesis, we crossed Cebpa_fl/fl mice with AdipoQ‐Cre mice, establishing mice with adipocyte‐specific knockout of *Cebpa* (Cebpa_ASKO). Here we report that Cebpa_ASKO mice have a near complete absence of gonadal WAT (gWAT), the largest vWAT depot in mice, due to a failure in gWAT development. In stark contrast, Cebpa_ASKO inguinal WAT (iWAT) is present in relatively normal quantities but has a drastically altered transcriptome and function. Lean Cebpa_ASKO mice also have multiple other metabolic phenotypes, including reduced lipolysis and plasma adiponectin, an inability to expand adipose depots when challenged with a high‐fat diet (HFD), increased brown adipose tissue (BAT) mass and lipid content, and increased hepatic triglycerides and plasma cholesterol. Taken together, these data illuminate a novel adipose depot‐specific role for C/EBP*α* and highlight its central role in whole body metabolism.

## 2 Methods

### 2.1 Animals

All animal studies were approved by Columbia University institutional IACUC committee, and all animals were housed in AAALAC‐accredited animal facilities. Cebpa_fl/fl (Jackson Labs strain #006447) and AdipoQ‐Cre + (Jackson Labs strain #010803) mice on the C57BL6/J background were acquired from Jackson Laboratories. These mice were crossed and maintained in‐house to generate experimental Cebpa_fl/fl; AdipoQ‐Cre + (*Cebpa* adipocyte‐specific knockout [Cebpa_ASKO]) and negative control Cebpa_fl/fl; AdipoQ‐Cre − (Cebpa_fl/fl) mice. Cebpa_ASKO and Cebpa_fl/fl mice for experiments were bred such that all Cebpa_fl/fl; AdipoQ‐Cre + and Cebpa_fl/fl; AdipoQ‐Cre − mice were true littermates. Mice were fed a chow diet unless on special diet. Mice were either postnatal days 1–14 (P1‐14) or 8–12 weeks old for all experiments unless otherwise indicated. For HFD feeding experiments, mice were fed a 60% kcal fat diet from Research Diets (D12492) ad libitum and maintained for 16–20 weeks. All in vivo findings were confirmed in multiple independent mouse cohorts, with data from a single representative male cohort shown in figures.

### 2.2 In Vivo Experiments

Blood for plasma lipid and ELISA measurements was collected retro‐orbitally from anesthetized 4‐h fasted or random‐fed mice with heparinized tubes. All blood samples were then spun at 1500*g* for 10 min at 4°C to separate plasma. Plasma cholesterol and triglycerides were measured in 4‐h fasted plasma via plate assay using Infinity Total Cholesterol and Infinity Triglycerides reagents (TR13421 and TR22421). Plasma from random‐fed mice or 4‐h fasted mice was used for all ELISA (adiponectin: Millipore‐Sigma EZMADP‐60K, leptin: Millipore‐Sigma EZML‐84K, PCSK9: R&D MPC900).

### 2.3 Statistics

Data were graphed using GraphPad Prism 10. Prism 10 was also used to perform two‐tailed Student's *t*‐tests to compare two groups. Multiple *t*‐tests were used when comparing two groups with multiple variables. Two‐way ANOVA with Tukey's post hoc was used when comparing three groups with multiple variables.

Further methods are detailed in the online Supporting Information.

## 3 Results

### 3.1 Cebpa_ASKO Mice Have Reduced gWAT Mass Due to Halted Development

**FIGURE 1:**
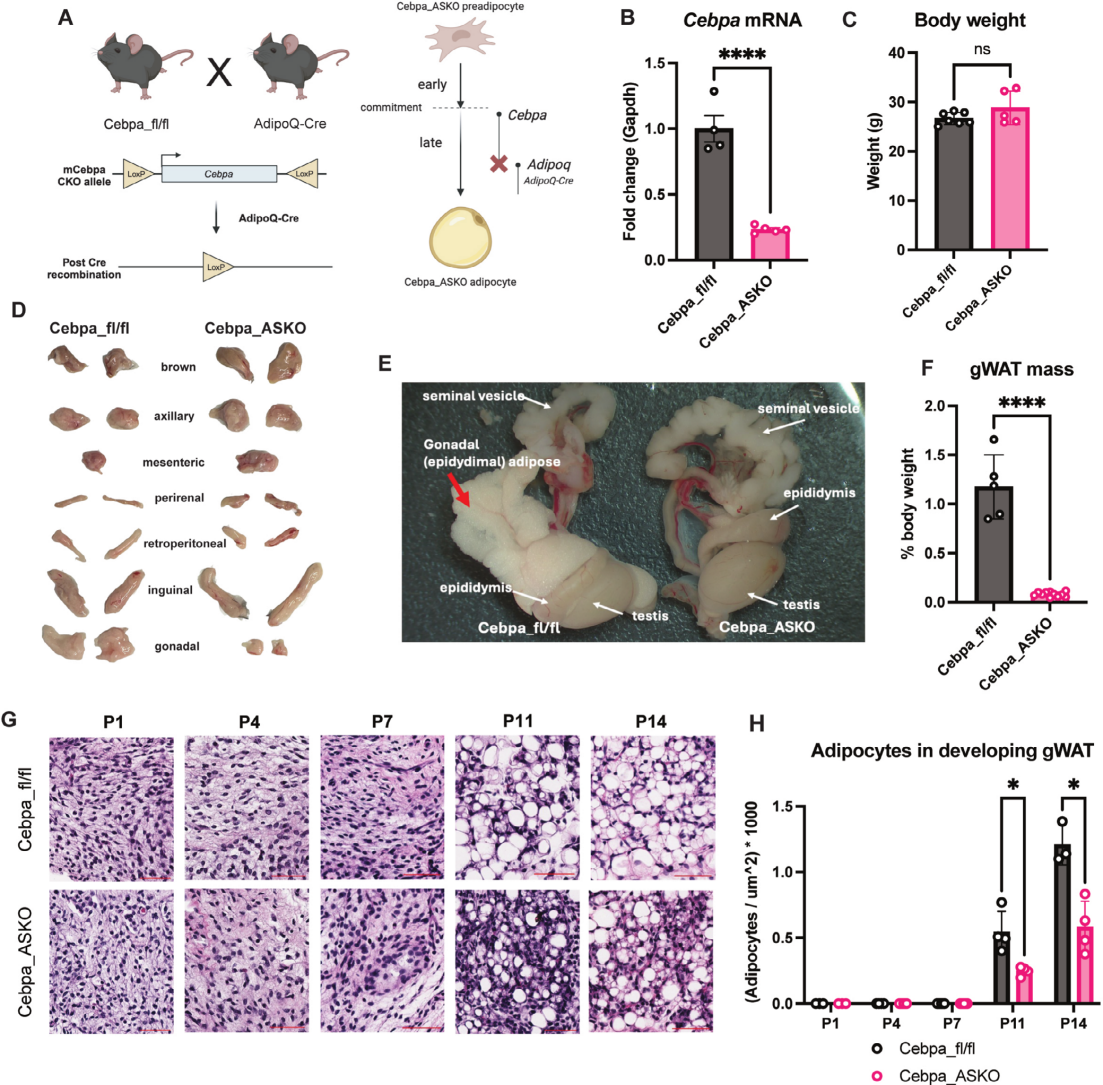
Cebpa_ASKO mice have altered gWAT development. (A) Schematic of Cebpa_ASKO mouse alleles and adipocyte differentiation knockout timeline. (B) Taqman qPCR for Cebpa in gWAT from male 10‐week‐old Cebpa_fl/fl and Cebpa_ASKO mice (*n* = 4–5). (C) Body weight in male 10– to 12‐week‐old Cebpa_fl/fl and Cebpa_ASKO mice (*n* = 5–7). (D) Macroscopic image of adipose tissue depots from male 10‐week‐old Cebpa_fl/fl and Cebpa_ASKO mice. (E) Macroscopic image of gWAT attached to gonads from male 10‐week‐old Cebpa_fl/fl and Cebpa_ASKO mice. (F) Mass of gWAT from male 10– to 12‐week‐old Cebpa_fl/fl and Cebpa_ASKO mice (*n* = 5–7). (G) H&E stain of developing gWAT appendage from Cebpa_fl/fl and Cebpa_ASKO neonates. (H) Identification and quantification of adipocytes by Adiposoft in one section of developing gWAT appendages. All mice were chow‐fed. Student's *t*‐test was used to analyze results (*p < 0.05, *****p < 0.0001). Schematic created with Biorender. [Color figure can be viewed at wileyonlinelibrary.com]

We generated Cebpa_ASKO mice by crossing Cebpa floxed mice (Cebpa_f/fl) with transgenic mice expressing Cre recombinase under control of the *Adipoq* promoter. As *Adipoq* is not expressed until late in the adipocyte differentiation process [11], Cebpa_ASKO mice represent a model of *Cebpa* knockout during late adipocyte differentiation (Figure 1A). We confirmed efficient knockout of *Cebpa* expression in 10‐week‐old Cebpa_ASKO gWAT (Figure 1B). Interestingly, 10‐week‐old Cebpa_ASKO mice do not display any outward phenotypes and have no significant difference in body weight compared to Cebpa_fl/fl controls (Figure 1C). However, upon sacrifice and tissue dissection, we immediately noticed a large reduction in gWAT mass in Cebpa_ASKO mice (Figure 1D), despite the normal appearance of other WAT depots (Figure S2). This finding was true in both male and female Cebpa_ASKO mice (Figure 1E,F, Figure S3). Together, these data clearly demonstrate a gWAT‐specific role for C/EBP*α* in WAT development.

gWAT is a visceral WAT depot that develops postnatally [14], unlike the subcutaneous iWAT that develops in utero. A previous study showed that male mouse gWAT develops during the first two postnatal weeks from an appendage underneath the corpus epididymis, with adipocytes first appearing around P7 [15]. Therefore, we hypothesized that Cebpa_ASKO gWAT development from the appendage diverges from Cebpa_fl/fl mice during this time. To test this, we performed a time‐course dissection of the developing appendage at postnatal day 1 (P1), P4, P7, P11, and P14 in both Cebpa_fl/fl and Cebpa_ASKO male mice. H&E staining in both groups revealed the appearance of adipocytes in the developing appendage between days P7 and P11 (Figure 1G). However, by P14, Cebpa_ASKO gWAT appendages have fewer lipid droplets than Cebpa_fl/fl gWAT appendages, consistent with disrupted adipocyte differentiation (Figure 1H). Thus, the severely decreased gWAT mass seen in 10‐week‐old Cebpa_ASKO mice is due to its halted development in the second perinatal week.

### 3.2 Cebpa_ASKO iWAT Adipocytes Develop Despite Loss of Cebpa

**FIGURE 2:**
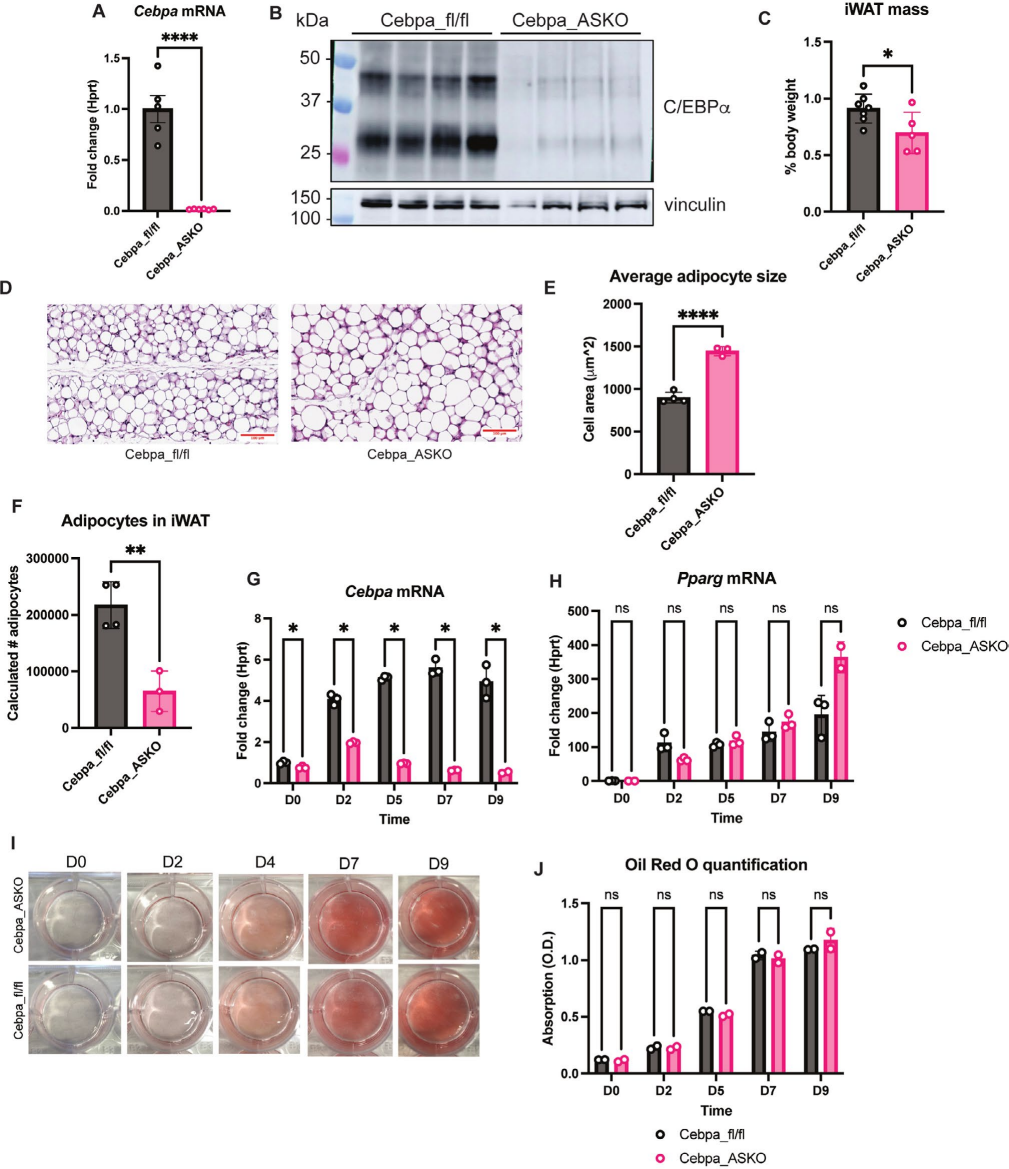
Cebpa_ASKO iWAT develops despite *Cebpa* loss. (A) Taqman qPCR for *Cebpa* in iWAT from Cebpa_fl/fl and Cebpa_ASKO mice (*n* = 5–7). (B) Western blot for C/EBP*α* on iWAT homogenates from 10‐week‐old male mice. (C) Mass of iWAT from 10‐ to 12‐week‐old male Cebpa_fl/fl and Cebpa_ASKO mice. (D) Representative H&E stain of iWAT from 10‐week‐old male mice. (E, F) Quantification of cell size by Adiposoft and an estimate of adipocyte number by the Goldrick formula (*n* = 3–4). (G) Taqman qPCR for *Cebpa* during differentiation in iWAT SVF. (H) Neutral lipid droplets during SVF‐derived adipocyte differentiation at D0, D2, D5, D7, and D9 were visualized by Oil Red O staining. (I) Quantification of extracted stained lipids from panel H (*n* = 2). (J) Leptin measured via ELISA in plasma of 4‐h fasted Cebpa_ASKO mice (*n* = 5–7). Student's *t*‐test was used to analyze results (*p < 0.05, **p < 0.01, ****p < 0.0001). [Color figure can be viewed at wileyonlinelibrary.com]

In contrast to gWAT, iWAT from adult Cebpa_ASKO mice appears grossly normal upon dissection despite *Cebpa* deletion (Figure 2A,B). Closer examination revealed that Cebpa_ASKO iWAT exhibits a small but significant reduction in mass compared to control mice (Figure 2C). Furthermore, Cebpa_ASKO iWAT adipocytes are larger than controls on average (Figure 2D,E, Figure S4), yet Cebpa_ASKO iWAT fat pads contain fewer adipocytes than control iWAT (Figure 2F), consistent with the smaller mass of the fat pad overall. To confirm that Cebpa_ASKO iWAT adipocytes can develop in a cell autonomous manner, we cultured and differentiated stromal vascular fraction cells (SVF) isolated from control and Cebpa_ASKO iWAT. In SVF from Cebpa_fl/fl mice, *Cebpa* exhibits a nearly sixfold increase in expression during differentiation as expected, while *Cebpa* expression briefly increases and then retreats to baseline levels in differentiating SVF from Cebpa_ASKO animals (Figure 2G), consistent with the expected timing of Cre expression. Conversely, *Pparg* expression remains unchanged between groups over the course of differentiation (Figure 2H), perhaps driving differentiation in the absence of *Cebpa* expression. We observed no difference in the capacity of Cebpa_fl/fl and Cebpa_ASKO iWAT SVF to develop lipid droplets over the course of differentiation (Figure 2I,J), consistent with the observed in vivo phenotype. These findings suggest that iWAT adipocytes are still able to accumulate lipid and develop the typical unilocular lipid droplet morphology of white adipocytes despite lacking *Cebpa* expression during late differentiation.

### 3.3 Cebpa_ASKO Mice Cannot Expand Adipose Depots Upon HFD Challenge

**FIGURE 3:**
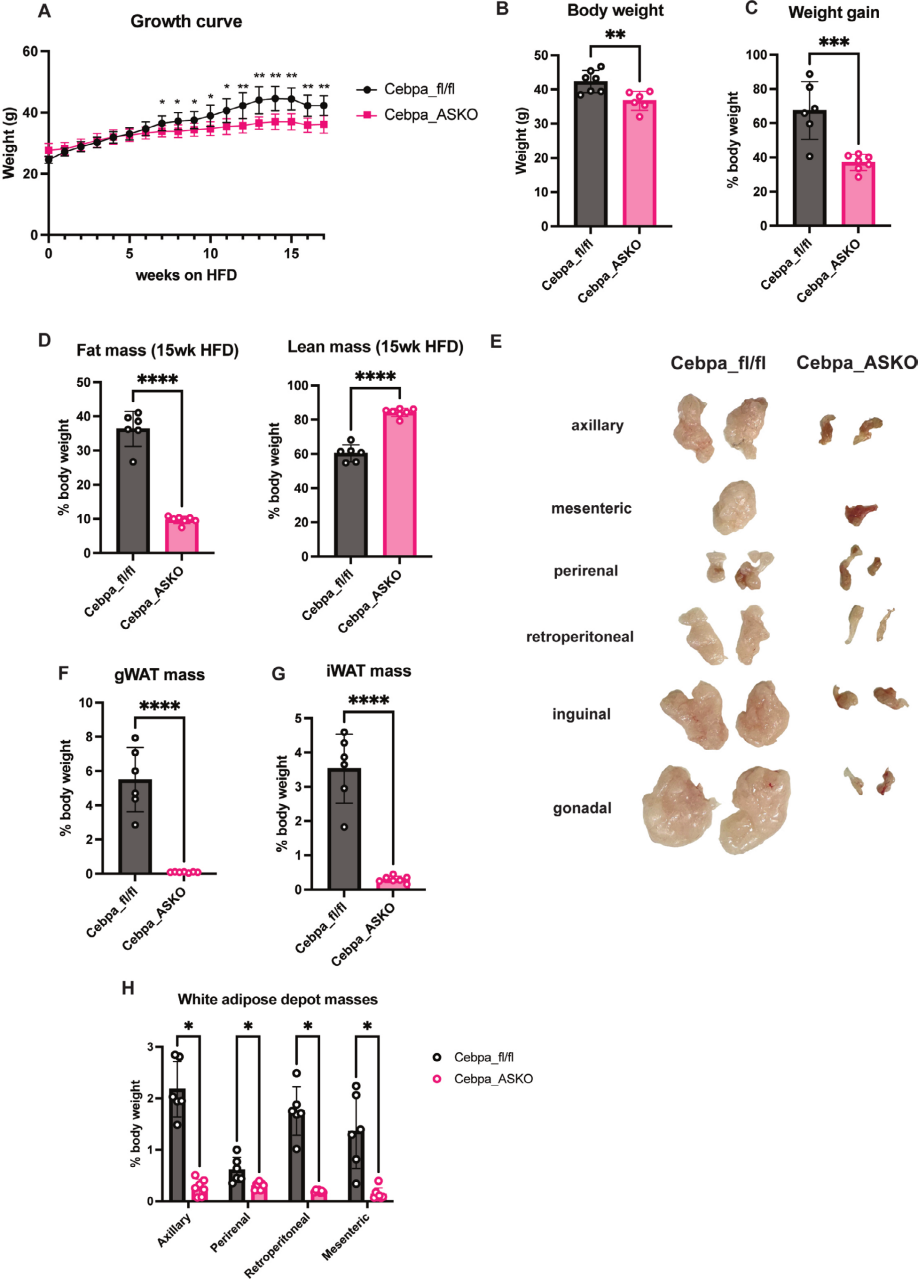
Cebpa_ASKO mice do not expand adipose on a high‐fat diet (HFD). (A) Growth curve of Cebpa_fl/fl and Cebpa_ASKO male mice fed HFD starting at 10–12 weeks of age. (B) Body weight of Cebpa_fl/fl and Cebpa_ASKO mice after 17 weeks of HFD feeding. (C) Weight gain in Cebpa_fl/fl and Cebpa_ASKO mice after 17 weeks of HFD feeding measured as percent change in body weight. (D) MRI measured fat mass and lean mass in a separate cohort of Cebpa_fl/fl and Cebpa_ASKO mice after 15 weeks of HFD feeding. (E) Macroscopic image of adipose depots of Cebpa_fl/fl and Cebpa_ASKO mice after 20 weeks of HFD feeding. (F–H) Masses of gWAT, iWAT, and other adipose depots from panel E reported by percent body weight (*n* = 6–7). Student's *t*‐test was used to analyze results (*p < 0.05, **p < 0.01, ***p < 0.001, ****p < 0.0001). [Color figure can be viewed at wileyonlinelibrary.com]

Given the specific defects in gWAT and iWAT in lean Cebpa_ASKO mice, we next asked if prolonged caloric excess would prompt adipose remodeling and overcome the overall reduction in adipose mass. We challenged Cebpa_fl/fl and Cebpa_ASKO mice with a 60% kcal HFD for 20 weeks and included aged‐matched chow‐fed controls. At baseline in control mice, *Cebpa* expression is higher in gWAT than iWAT, while 20 weeks of HFD feeding induces an increase in iWAT *Cebpa* expression and a decrease in gWAT, consistent with depot‐specific roles for C/EBP*α* (Figure S5). We found that HFD‐fed Cebpa_ASKO mice gain significantly less weight than controls (Figure 3A–C). MRI measurements showed that Cebpa_ASKO mice had less fat mass and more lean mass than controls at 15 weeks of HFD feeding (Figure 3D), suggesting that the inability of Cebpa_ASKO mice to gain weight by HFD challenge is due to disrupted adipose expansion. Consistent with this, Cebpa_ASKO mice exhibited a complete absence of adipose depot expansion after 20 weeks on HFD, in stark contrast to the normal expansion of WAT depots in Cebpa_fl/fl mice (Figure 3E,H). Specifically, Cebpa_ASKO gWAT remained nearly completely absent, and Cebpa_ASKO iWAT did not expand (Figure 3F,G). These data suggest that all mature WAT depots require *Cebpa* expression for expansion via HFD‐induced hyperplasia or hypertrophy. Furthermore, iWAT and axillary WAT exhibit significant reductions in mass in Cebpa_ASKO aged or HFD‐fed mice, suggesting that C/EBP*α* is not only required for HFD‐induced expansion, but also simple maintenance of adipose tissue during aging (Figure S6). These data support the idea that *Cebpa* expression is more crucial for continuous iWAT growth and expansion over time or with HFD feeding compared to other depots, despite the seemingly gWAT‐specific phenotype at baseline.

### 3.4 Cebpa_ASKO iWAT Adipocytes Have Altered Transcriptomes and Function

**FIGURE 4:**
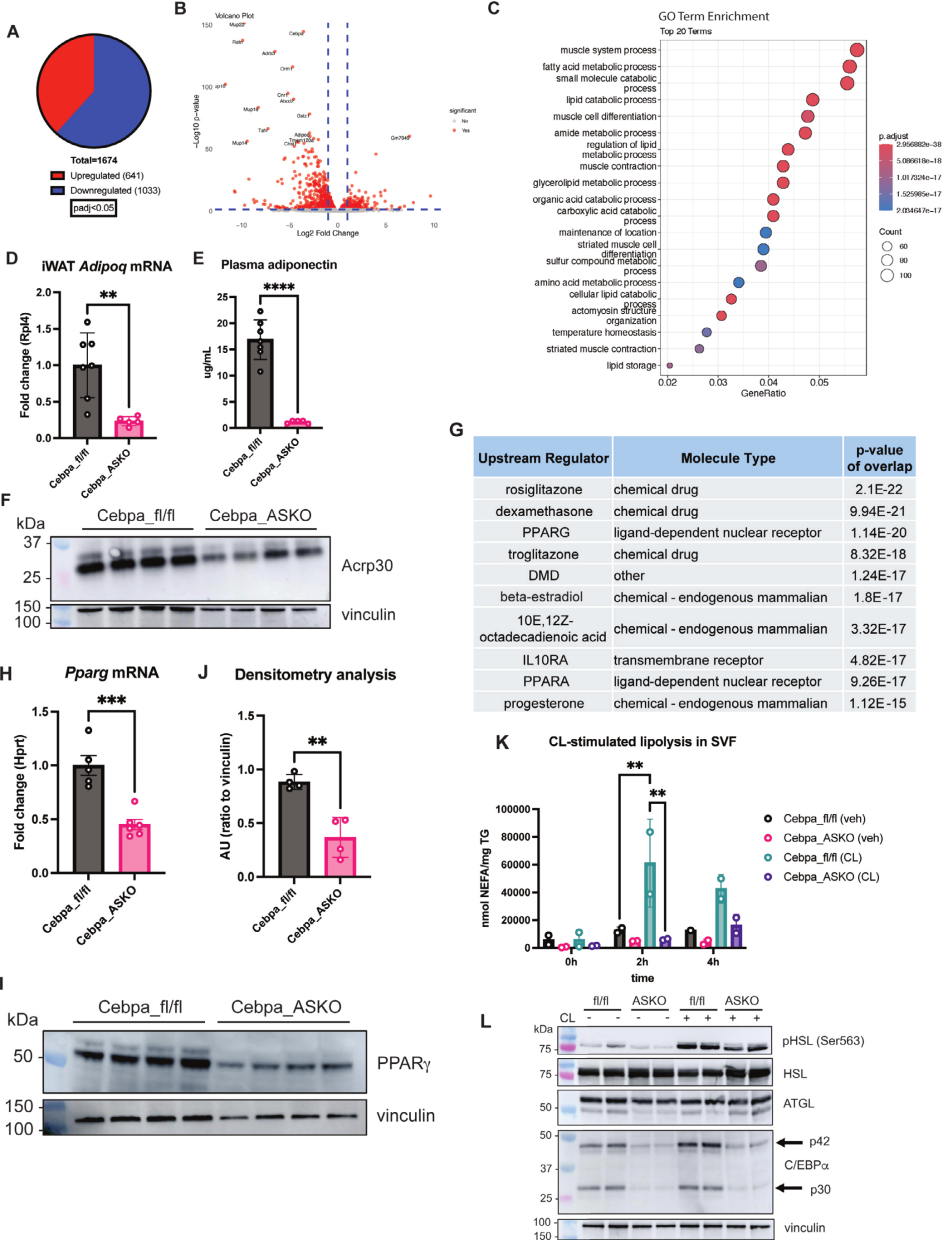
Cebpa_ASKO iWAT is dysfunctional. (A) Number of differentially expressed genes in bulk RNA‐seq of control and Cebpa_ASKO iWAT total tissue RNA. (B) Volcano plot of DESeq2 analysis of bulk RNA‐seq data from Cebpa_fl/fl and Cebpa_ASKO iWAT (*n* = 3). (C) Gene set enrichment analysis for GO terms from bulk iWAT RNA‐seq data. (D) Taqman qPCR for *Adipoq* in iWAT from Cebpa_fl/fl and Cebpa_ASKO mice (*n* = 5–7). (E) Adiponectin measured via ELISA in plasma of 4‐h fasted Cebpa_ASKO mice (*n* = 5–7). (F) Adiponectin (Acrp30) protein levels measured via Western blot in lysates from Cebpa_fl/fl and Cebpa_ASKO iWAT. (G) Identification of upstream regulators most likely to contribute to differences in gene expression using Ingenuity Pathway Analysis. (H) Taqman qPCR for *Pparg* in whole iWAT (*n* = 5–6). (I) Western blot analysis of PPARγ in homogenates of whole iWAT (*n* = 4). (J) Densitometry analysis of panel I, normalized to vinculin. (K) Nonesterified fatty acid concentration measured in media from iWAT SVF‐derived adipocytes treated with vehicle or CL‐316,243, normalized to triglyceride (TG) mass of each sample (*n* = 2). (L) Western blot analysis of lipolysis markers in homogenates of SVF‐derived adipocytes from panel G after 2 h of stimulation. All mice were 10–12 weeks old and chow‐fed. Student's *t*‐test was used to analyze results (**p < 0.01, ***p < 0.001, ****p < 0.0001). [Color figure can be viewed at wileyonlinelibrary.com]

The observation that Cebpa_ASKO iWAT depots could not expand after prolonged HFD challenge suggests adipocyte dysfunction, despite their somewhat normal morphology. To further investigate this, we performed bulk RNA‐seq on whole iWAT from lean, 10‐week‐old Cebpa_fl/fl and Cebpa_ASKO mice (Figure 4A,B). Gene set enrichment analysis (GSEA) revealed changes in multiple lipid metabolism processes including fatty acid metabolism and lipid catabolism, consistent with disruption of the canonical adipocyte function of storing and mobilizing lipids (Figure 4C). Interestingly, although iWAT adipocytes have unilocular white adipocyte morphology and iWAT SVF can differentiate, Cebpa_ASKO iWAT has decreased expression of numerous genes important for adipocyte differentiation (*Pparg*, *Zfp423*, *Fabp4*, *Adipoq*) (Figure S7). This is consistent with the known role for C/EBP*α* in transcriptional regulation of differentiation but suggests that a lower expression of these genes is tolerated during iWAT differentiation. Validating our RNA‐seq results, *Adipoq* expression is decreased in Cebpa_ASKO iWAT (Figure 4D–F), and Cebpa_ASKO mice also have significantly lower circulating adiponectin compared to controls (Figure 4E), consistent with prior literature showing that *Adipoq* is a downstream target of C/EBP*α* along with PPAR*γ* [16, 17, 18]. However, considering that adiponectin itself is a commonly used marker for mature adipocytes [19], reduced adiponectin circulation and expression again pointed toward disruption of normal adipocyte function in Cebpa_ASKO animals.

Ingenuity pathway analysis revealed that the most significantly changed upstream regulatory networks in Cebpa_ASKO iWAT were those regulated by PPAR*γ* or PPAR*γ* agonists, suggesting that decreased PPAR*γ* activity is contributing to the reduced function (Figure 4G). *Pparg* expression and protein levels are indeed decreased in whole Cebpa_ASKO iWAT (Figure 4H–J), pointing toward a decrease in PPAR*γ* activity in Cebpa_ASKO iWAT that is consistent with the described positive regulatory loop of C/EBP*α* and PPAR*γ* [12, 20]. It is possible that the decrease in PPAR*γ* expression and activity is driving the dysfunction in Cebpa_ASKO adipocytes in conjunction with the loss of *Cebpa*, while the residual *Pparg* expression may be sufficient to drive iWAT development in Cebpa_ASKO mice.

Our RNA‐seq results also indicated decreased expression of lipolysis genes in Cebpa_ASKO iWAT compared to controls (Figure S7), including *Pnpla2*, *Lipe*, and *Mgll*. Lipolysis is a characteristic function of adipocytes and is largely recognized to be regulated at the protein level, so decreased transcription of these genes may not result in functional changes [21]. Therefore, we investigated lipolysis in SVF‐derived adipocytes from Cebpa_ASKO iWAT. Upon stimulating lipolysis with CL‐316,243 in differentiated SVF‐derived iWAT adipocytes, we found that release of nonesterified fatty acids was decreased in Cebpa_ASKO cells compared to Cebpa_fl/fl cells (Figure 4K). Additionally, Western blot analysis showed decreased protein levels of lipolysis markers such as phosphorylated Ser563 in HSL in the Cebpa_ASKO lysates (Figure 4L). These data confirmed that Cebpa_ASKO iWAT adipocytes have decreased lipolysis function compared to Cebpa_fl/fl adipocytes. Taken together, Cebpa_ASKO iWAT demonstrates multiple aspects of dysfunction, including an altered transcriptome, reduced adipokine production, and decreased lipolysis, despite the tissue having a somewhat normal appearance.

### 3.5 Cebpa_ASKO Mice Have Whiter BAT

**FIGURE 5:**
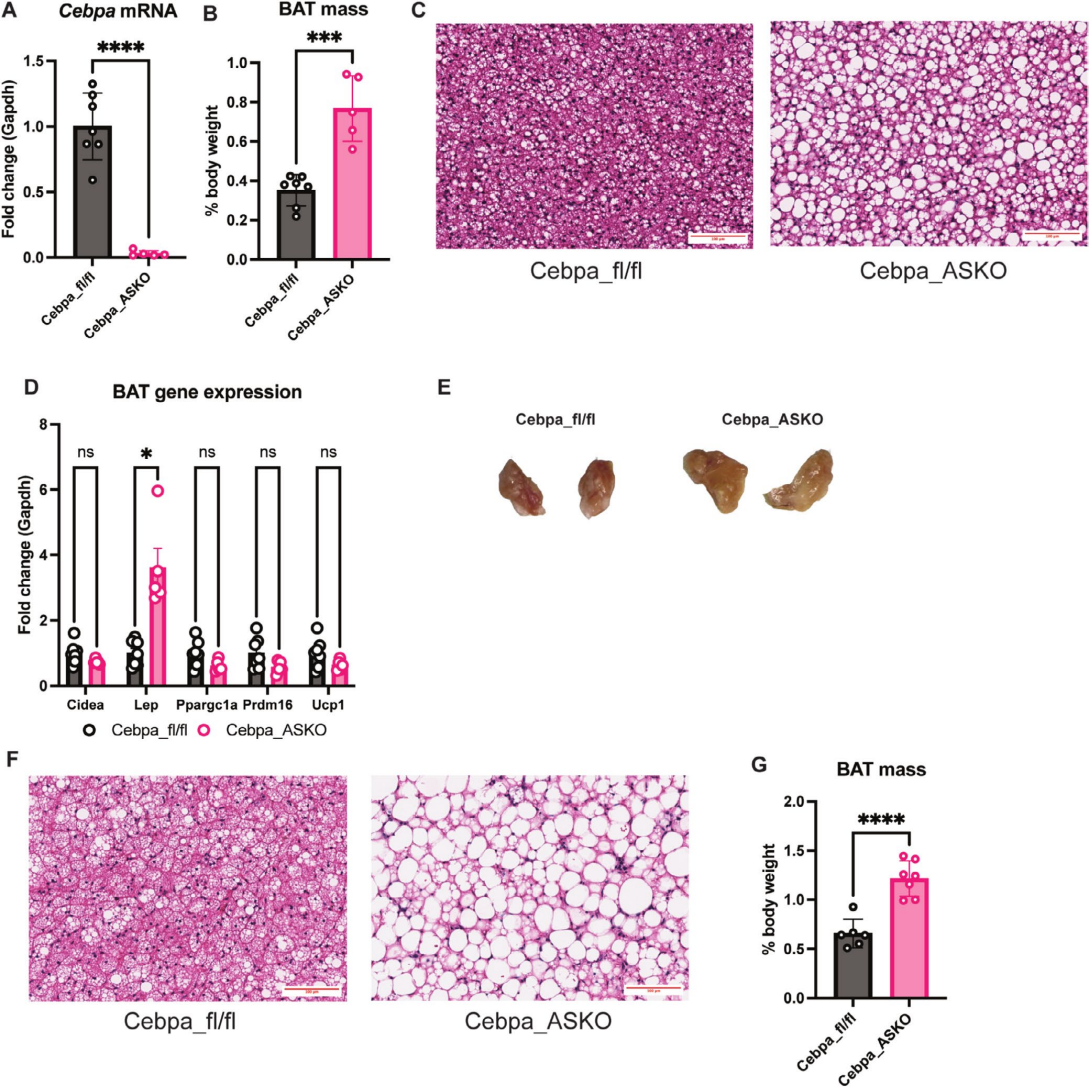
Cebpa_ASKO mice have whitened BAT. (A) Taqman qPCR for *Cebpa* in RNA extracted from whole BAT (*n* = 5–7). (B) BAT mass reported as percentage of body weight in Cebpa_fl/fl and Cebpa_ASKO mice. (C) Representative H&E stain of BAT from Cebpa_fl/fl and Cebpa_ASKO mice. (D) Taqman qPCR for *Cidea*, *Lep*, *Ppargc1a*, *Prdm16*, and *Ucp1* expression in RNA extracted from whole BAT (*n* = 5–7). (E) Representative H&E stain of BAT from Cebpa_fl/fl and Cebpa_ASKO mice fed HFD for 20 weeks. (F) Macroscopic image of BAT from panel E. (G) BAT mass reported as percentage of body weight in Cebpa_fl/fl and Cebpa_ASKO mice after 20 weeks of HFD feeding. Male mice were 10–12 weeks old and chow‐fed unless otherwise noted. Student's *t*‐test was used to analyze results (*p < 0.05, ***p < 0.001, ****p < 0.0001). [Color figure can be viewed at wileyonlinelibrary.com]

Given that the AdipoQ‐Cre transgene is expressed in BAT as well, we found that Cebpa_ASKO mice have efficient *Cebpa* knockout in their BAT (Figure 5A). Cebpa_ASKO BAT has significantly increased mass (Figure 5B), and H&E staining revealed the Cebpa_ASKO BAT to have excess lipid droplet content (Figure 5C). Consistent with this “whitening,” there is a downward trend in the expression of typical brown adipocyte markers (*Ucp1*, *Cidea*, *Ppargc1a*) that does not reach significance. Additionally, expression of leptin, an indicator of whitening and white adipocyte identity [22], was increased in Cebpa_ASKO BAT (Figure 5D), reflecting the appearance of unilocular lipid droplets, a trait of white adipocytes. Mice challenged with 20 weeks of HFD feeding showed progression of increased BAT mass and worsening lipid accumulation (Figure 5E–G). These findings suggest a potential role for C/EBP*α* in BAT lipid metabolism or storage [13].

### 3.6 Cebpa_ASKO Mice Have Hypertrophic Livers

**FIGURE 6:**
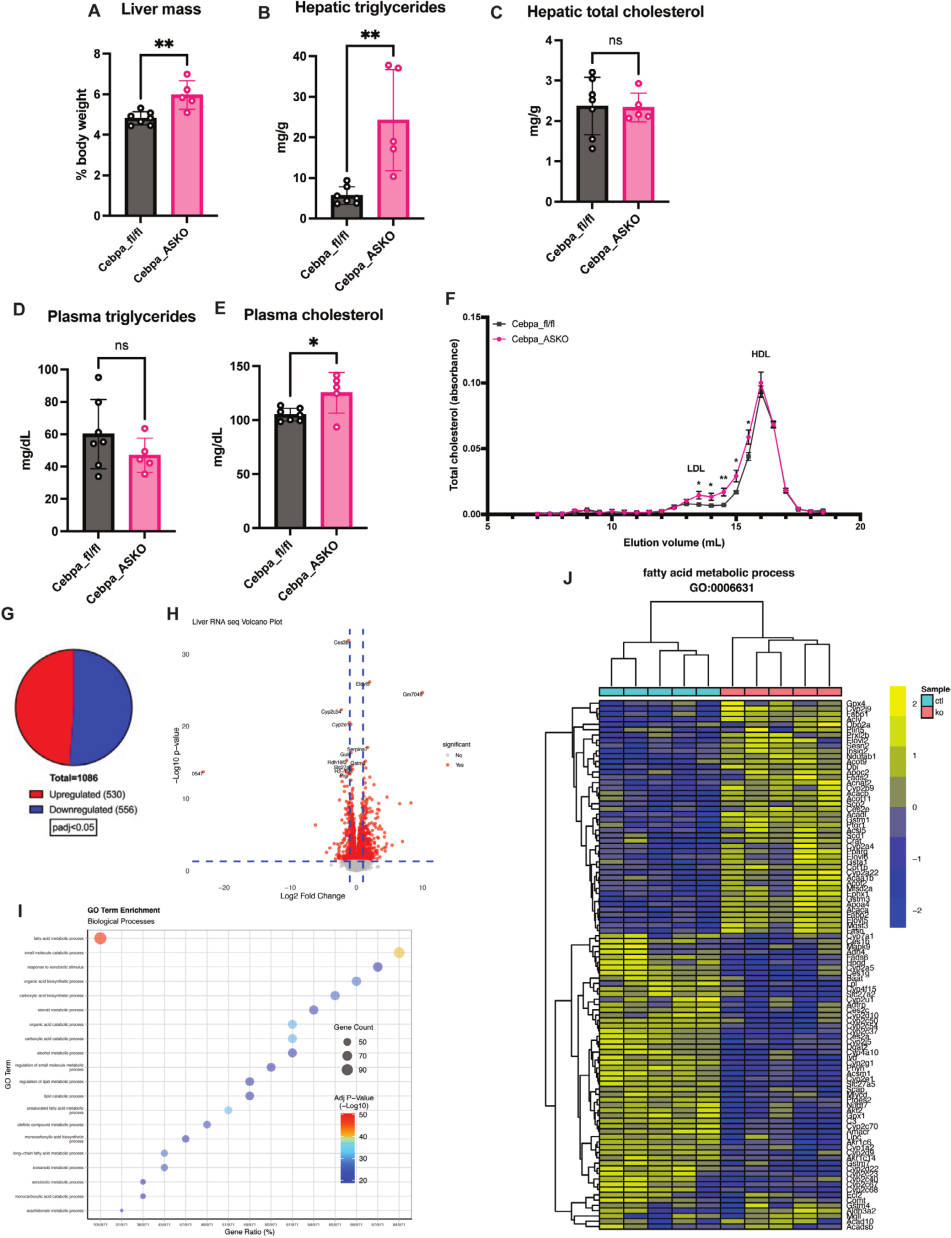
Cebpa_ASKO mice have increased lipid accumulation in their livers. (A) Liver mass reported as percentage of body weight from Cebpa_fl/fl and Cebpa_ASKO mice. (B) Hepatic triglycerides and (C) cholesterol measured in liver homogenates (*n* = 5–7). (D) Plasma triglycerides and (E) cholesterol were measured in 4‐h fasted mice (*n* = 5–7). (F) Cholesterol FPLC profile of pooled plasma (*n* = 5–6) from 4‐h fasted male Cebpa_fl/fl and Cebpa_ASKO mice. (G) Pie chart of the number of differentially expressed genes in bulk RNA‐seq. (H) Volcano plot of DESeq2 analysis of bulk RNA‐seq data from Cebpa_fl/fl and Cebpa_ASKO livers (*n* = 5). (I) Gene set enrichment analysis for GO terms from RNA‐seq data. (J) Heat map of expression of genes clustered in the fatty acid metabolic process GO term from bulk RNA‐seq. All mice were 10–12 weeks old and chow‐fed. Student's *t*‐test was used to analyze results (*p < 0.05, **p < 0.01). [Color figure can be viewed at wileyonlinelibrary.com]

In both mice and humans, partial and complete lipodystrophies impact hepatic lipid metabolism [23, 24]. As our Cebpa_ASKO mice have partial lipodystrophy shown by reduced WAT mass and altered WAT lipid metabolism, we investigated the livers of Cebpa_ASKO mice. Lean Cebpa_ASKO mice have significantly larger livers than Cebpa_fl/fl mice (Figure 6A). They also have increased hepatic triglycerides, although hepatic cholesterol remains unchanged (Figure 6B,C). Cebpa_ASKO mice also have increased plasma cholesterol, with no change in plasma triglycerides compared to controls (Figure 6D,E). FPLC analysis revealed that the increased plasma cholesterol in Cebpa_ASKO mice is in the LDL fraction (Figure 6F), while also demonstrating a reduction in VLDL triglyceride content not captured in whole plasma analysis (Figure S8A). These phenotypes are all further exacerbated in Cebpa_ASKO mice challenged with HFD feeding (Figure S9). Given the increase in LDL cholesterol, we investigated the well‐known LDLR/PCSK9 cholesterol metabolic pathway [25]. We found no change in both circulating plasma PCSK9 levels and hepatic protein levels of LDLR (Figure S8B,C). These indicate that the increase in plasma cholesterol in Cebpa_ASKO mice is independent of the PCSK9/LDLR pathway.

To further investigate the effect of the multiple adipose depot phenotypes on hepatic lipid metabolism in Cebpa_ASKO mice, we performed bulk RNA‐seq on livers from lean, 10– to 12‐week‐old Cebpa_fl/fl and Cebpa_ASKO (Figure 6G,H). GSEA showed fatty acid metabolism to be the most significant gene ontology signature in Cebpa_ASKO livers (Figure 6I), with upregulation of fatty acid metabolism genes such as *Fasn*, *Scd1*, and *Elovl6*, aligning with the increase in hepatic triglycerides (Figure 6J). Further validation via qPCR was consistent with these data and points to increased hepatic triglyceride synthesis (Figure S10). These findings suggest that increased hepatic triglyceride deposition and synthesis in Cebpa_ASKO livers may be driving altered hepatic lipid and lipoprotein metabolism.

## 4 Discussion

C/EBP*α* has been well described as a master transcriptional regulator of adipogenesis. Prior studies of mice harboring global germline deletion of *Cebpa* demonstrate a complete lack of WAT development, underscoring the importance of C/EBP*α* for normal WAT development. Interestingly, GWAS have identified SNPs near *CEBPA* that associate with metrics of visceral adiposity such as WHRadjBMI, suggesting that C/EBP*α* plays depot‐specific roles in WAT development, yet there is no prior experimental evidence supporting this. Here, we report a novel model of adipocyte‐specific *Cebpa* knockout that dramatically reduces gWAT mass, the largest visceral adipose depot in mice, while leaving iWAT somewhat intact, demonstrating a gWAT‐specific role for C/EBP*α* in WAT development. These data are the first in vivo validation of the 19q13 GWAS association with WHRadjBMI and suggest that *CEBPA* is a causal gene at this locus.

The *Cebpa* gene lies in the 19q13 GWAS locus, in which SNPs have been repeatedly associated with metrics of adiposity such as BMI, WHR, WHRadjBMI, visceral adipose tissue volume, and body fat percentage [9, 10, 26] and other metabolic traits including circulating adiponectin and plasma total cholesterol [27, 28]. There are three genes in the 19q13 GWAS locus: *CEBPA*, *PEPD*, and *CEBPG* (Figure S1), with most SNPs annotated to *PEPD* given that some GWAS SNPs lie in the first *PEPD* intron [9, 29, 30, 31]. A recent report demonstrated that macrophage *Pepd* can regulate adipose fibrosis in mice [32], but experimental evidence supporting *Pepd* or *Cebpg* as the causal gene(s) in this locus is scant, particularly in comparison to the body of literature implicating *Cebpa* in many of the associated phenotypes. Interestingly, many GWAS SNPs in this region demonstrate eQTLs with *PEPD* expression in human adipose tissue per the GTEx consortium, while *CEBPA* is completely devoid of any adipose eQTLs despite its well‐characterized role in adipose development [33]. Given the multitude of metabolic GWAS associations in this locus, further study is warranted to determine the causal genes and biological mechanisms underlying those associations. Despite the lack of eQTLs, the data presented in this manuscript support the notion that *CEBPA* is one of the causal genes in the 19q13 GWAS locus.

Although past studies have investigated the effects of acutely induced adipocyte‐specific *Cebpa* knockout, none has reported adipose depot‐specific effects. Wang et al. reported that inducing *Cebpa* knockout via doxycycline‐inducible Cre from embryonic day 11 through postnatal day 16 did not affect gWAT development or mass [34]. Unlike that model, our Cebpa_ASKO mice have a knockout of *Cebpa* driven by the constitutively active AdipoQ‐Cre transgene, and we suspect that any phenotypic differences between the models must be related to the method of knockout. Our use of the AdipoQ‐Cre model introduces some complexity in understanding when *Cebpa* knockout occurs, as *Adipoq* is expressed only in the late stages of adipogenesis, and its transcription is driven by C/EBP*α*, which is active in earlier stages [17, 35]. Therefore, our interpretation of the Cebpa_ASKO mouse is that *Cebpa* would be expressed in preadipocytes in early differentiation but then silenced near maturation, and we confirmed this with an in vitro iWAT SVF time‐course experiment (Figure 2G–I). Since preadipocytes in all Cebpa_ASKO WAT depots presumably express *Cebpa* early in the adipogenic program, the lack of gWAT in Cebpa_ASKO mice suggests that gWAT is more sensitive to loss of C/EBP*α* in mid‐ to late‐stage differentiation than other depots.

The lack of gWAT in Cebpa_ASKO mice aligns with our understanding of C/EBP*α* as a critical regulator of adipose development, whereas the maintenance of iWAT in those same mice defies expectations. In mice, these depots arise from distinct progenitor cell populations: iWAT develops from the *Prrx1* lineage while gWAT develops from *Wt1*‐expressing cells [36, 37, 38]. Previous studies have identified a particularly adipogenic cluster of adipocyte progenitor cells (APCs), sometimes termed “committed preadipocytes,” that are present in both adult and developing iWAT and male gWAT [39, 40, 41]. Interestingly, this subpopulation expresses *Pparg*, *Adipoq*, *Fabp4*, and *Plin1* prior to lipid droplet formation, and it appears fibroblast‐like in vitro [39, 41, 42, 43]. When isolated from embryonic iWAT or perinatal gWAT and cultured in vitro, these cells accumulate lipid and differentiate more efficiently than other populations of progenitor cells [41, 43]. A possible explanation for the Cebpa_ASKO phenotype is that there are more committed preadipocytes in developing mouse iWAT than gWAT, and these cells are more tolerant to loss of *Cebpa* during differentiation. As we also observe reductions in PPAR*γ* mRNA and protein in Cebpa_ASKO iWAT, this developmental threshold may involve the entire C/EBP*α*‐PPAR*γ* feedback loop. Consistent with this idea, iWAT SVF‐derived adipocytes develop completely normally ex vivo and exhibit no changes in *Pparg* expression during differentiation (Figure 2H). It is possible that this difference or PPAR*γ* agonism masks the effects of C/EBP*α* deficiency in our iWAT SVF‐derived adipocytes (Figure 2I,J), but that this would not be the case in gWAT SVF‐derived adipocytes, highlighting threshold differences in the two depots. Further studies identifying the specific altered APC populations in Cebpa_ASKO developing gWAT will shed light on the mechanisms governing these developmental thresholds. Overall, our findings add to the growing body of literature demonstrating that scWAT and vWAT have different developmental programs and functions despite both being WAT.

Unlike the two main WAT pads, Cebpa_ASKO BAT is larger and lipid‐laden, a phenotype that worsens upon extended HFD feeding. The appearance of large lipid droplets in Cebpa_ASKO BAT is consistent with results from previously reported *Cebpa* knockout models [13, 34]. BAT hypertrophy was also seen in the transgenic global *Cebpa* knockout mice [13], confirming that C/EBP*α* is not necessary for BAT development. Notably, previous data from the inducible adipocyte‐specific *Cebpa* knockout model also show lipid‐laden BAT despite no change in WAT mass, suggesting that the whitened appearance of Cebpa_ASKO BAT is not due to partial lipodystrophy [34]. Therefore, it is possible that C/EBP*α* acts as a brake on whitening of brown adipocytes during development, leading to whitening upon *Cebpa* loss. The Cebpa_ASKO BAT may also be dysfunctional allowing for lipid accumulation, a possibility reflected in the downward trend of brown adipocyte gene expression. C/EBP*α* clearly regulates lipid accumulation in BAT, but further investigation is required to understand the exact mechanisms at play.

Finally, Cebpa_ASKO mice also exhibit numerous alterations in hepatic physiology and function, including elevated hepatic triglycerides and plasma LDL cholesterol, and multiple transcriptional changes in hepatic fatty acid metabolism genes. It is plausible that these traits are a result of excess lipid deposition in the liver due to the lack of gWAT in these mice. Furthermore, significant changes in iWAT function, including reduced adipokine secretion and lipolysis, could be affecting liver function via altered cross talk to the liver, although reduced lipolysis in Cebpa_ASKO mice does not align with increased hepatic triglyceride content. Given the ongoing interest in the relationship between vWAT and cardiometabolic traits [6], the mechanisms governing the increased hepatic triglyceride content and plasma LDL cholesterol levels in Cebpa_ASKO mice are the focus of ongoing investigations.

Altogether, these data demonstrate that adipocyte C/EBP*α* is selectively required for gWAT development in mice. This finding validates the GWAS association of SNPs near *CEBPA* at the 19q13 locus with adiposity metrics such as WHR and suggests that C/EBP*α* contributes to determining adipose distribution in humans. In humans, recent attempts to create a single‐cell atlas of human adipose tissue have shown no difference in the expression of *CEBPA* between visceral and subcutaneous depots (with low expression overall), underscoring the need for further investigation into the mechanisms underlying the genetic link between *CEBPA* and WHR [44, 45]. As increased WHR has been well documented as tracking negatively with metabolic traits, such as an increased risk for heart disease, understanding the genetic contributions of *CEBPA* and its role in vWAT development brings us one step closer to curbing increasing WHR in the population.

## Funding

This work was supported by NIH grants T32DK007647 (K.Y.H.), T32DK007328 (K.Y.H.), R01DK134026 (R.C.B.), R01HL141745 (R.C.B.), P30DK026887 (R.C.B.), and R01DK134026‐02S1 (J.G.P.) and American Heart Association grants 23PRE1020947 (K.Y.H.) and 23TPA1077613 (R.C.B.).

## Conflicts of Interest

The authors declare no conflicts of interest.

## Supporting information


**Supplementary Figure S1:** The *CEBPA* GWAS locus. LocusZoom plot of SNPs at the 19q13 locus associated with waist to hip ratio adjusted for BMI from the GIANT consortium [55].
**Supplementary Figure S2:** Cebpa_ASKO mice have no change in other WAT depot masses. (A) Axillary, mesenteric, perirenal, and retroperitoneal adipose depot masses as percent body weight in male mice at 10–12 weeks of age (*n* = 3). All mice were chow‐fed. Student's *t*‐test was used to analyze results.
**Supplementary Figure S3:** Female Cebpa_ASKO mice have reduced gWAT mass. (A) TaqMan qPCR of *Cebpa* expression in (iwat) and BAT from 10‐ to 12‐week‐old female Cebpa_fl/fl and Cebpa_ASKO mice (*n* = 6–7). (B) Body weight at 10–12 weeks of female Cebpa_fl/fl and Cebpa_ASKO mice (*n* = 10). (C–E) gWAT, iWAT, and liver masses as percent body weight at 10–12 weeks of age (*n* = 10). All mice were chow‐fed. Student's *t*‐test was used to analyze results (**p < 0.01, ****p < 0.0001).
**Supplementary Figure S4:** Cebpa_ASKO mice have larger iWAT adipocytes. (A) Size distribution of adipocytes calculated using the Adiposoft FIJI plugin from representative iWAT H&E images of 10– to 12‐week‐old mice (*n* = 3). All mice were chow‐fed. Student's *t*‐test was used to analyze results (*p < 0.05, **p < 0.01).
**Supplementary Figure S5:** Cebpa has increased expression in expanding iWAT compared to gWAT. Taqman qPCR for Cebpa in RNA extracted from whole iWAT or gWAT (*n* = 5–7) in Cebpa_fl/fl mice at 10 weeks of age (chow‐fed), after 20 weeks of additional chow feeding, or after 20 weeks of additional HFD feeding. Student's *t*‐test was used to analyze results (*p < 0.05, **p < 0.01).
**Supplementary Figure S6:** WAT mass in Cebpa_ASKO mice reduces over time regardless of diet. Masses of white adipose depots expressed as percentage of body weight (*n* = 7–11) in male Cebpa_ASKO mice at 10 weeks of age (chow‐fed), after 20 weeks of additional chow feeding, or after 20 weeks of additional HFD feeding. Student's *t*‐test was used to analyze results (*p < 0.05, **p < 0.01, ***p < 0.001).
**Supplementary Figure S7:** Cebpa_ASKO iWAT has changes in expression of adipocyte differentiation genes. Heatmap of expression of genes clustered in the fat cell differentiation GO term from bulk RNA‐seq data (*n* = 3). All mice were chow‐fed.
**Supplementary Figure S8:** Cebpa_ASKO mice exhibit normal hepatic LDLR pathway function. (A): Triglyceride (TG) FPLC profile of pooled plasma (*n* = 5–6) from 4‐h fasted male Cebpa_fl/fl and Cebpa_ASKO mice. (B) Plasma PCSK9 levels measured via ELISA in 4‐h fasted mice (*n* = 5). (C) Western blot analysis of hepatic LDLR protein levels. Student's *t*‐test was used to analyze results (****p < 0.0001).
**Supplementary Figure S9:** High fat diet feeding exacerbates the lipid deposition in livers of Cebpa_ASKO mice. (A) Liver mass measured in HFD‐fed Cebpa_fl/fl and Cebpa_ASKO mice. (B) Macroscopic image of livers from HFD‐fed Cebpa_fl/fl and Cebpa_ASKO mice. (C). Representative images from H&E‐stained livers. All mice were fed with HFD for 20 weeks. Student's *t*‐test was used to analyze results (****p < 0.0001).
**Supplementary Figure S10:** Cebpa_ASKO mice have increased expression of hepatic triglyceride synthesis genes. (A) Taqman qPCR for Srebf1, Acaca, Scd1, Fasn, Dgat1, and Dgat2 in RNA extracted from whole livers of chow‐fed male Cebpa_fl/fl and Cebpa_ASKO mice at 10 weeks of age (*n* = 5–6). (B) Taqman qPCR for Ppar, Ppargc1a, Acox1, Cpt1a, Ehhadh, and Acadl in RNA extracted from whole livers of chow‐fed male Cebpa_fl/fl and Cebpa_ASKO mice at 10 weeks of age (*n* = 5–6). Student's *t*‐test was used to analyze results (*p < 0.05).

## Data Availability

The data that support the findings of this study are openly available in Gene Expression Omnibus at https://www.ncbi.nlm.nih.gov/geo/, reference number GSE302944.
