# Supplementary material for: C/EBP α is Essential for Gonadal but Not Inguinal White Adipose Tissue Formation in Mice
Source: Obesity (Silver Spring). 2026 Feb 11;34(4):882–95. doi: 10.1002/oby.70142 (PMC13032053; doi:10.1002/oby.70142)
Supplement: Supplementary file 1 — Supplementary Figure S1: The CEBPA GWAS locus. LocusZoom plot of SNPs at the 19q13 locus associated with waist to hip ratio adjusted for BMI from the GIANT consortium [55]. Supplementary Figure S2: Cebpa_ASKO mice have no change in other WAT depot masses. (A) Axillary, mesenteric, perirenal, and retroperitoneal adipose depot masses as percent body weight in male mice at 10–12 weeks of age (n = 3). All mice were chow‐fed. Student's t‐test was used to analyze results. Supplementary Figure S3: Female Cebpa_ASKO mice have reduced gWAT mass. (A) TaqMan qPCR of Cebpa expression in (iwat) and BAT from 10‐ to 12‐week‐old female Cebpa_fl/fl and Cebpa_ASKO mice (n = 6–7). (B) Body weight at 10–12 weeks of female Cebpa_fl/fl and Cebpa_ASKO mice (n = 10). (C–E) gWAT, iWAT, and liver masses as percent body weight at 10–12 weeks of age (n = 10). All mice were chow‐fed. Student's t‐test was used to analyze results (**p < 0.01, ****p < 0.0001). Supplementary Figure S4: Cebpa_ASKO mice have larger iWAT adipocytes. (A) Size distribution of adipocytes calculated using the Adiposoft FIJI plugin from representative iWAT H&E images of 10– to 12‐week‐old mice (n = 3). All mice were chow‐fed. Student's t‐test was used to analyze results (*p < 0.05, **p < 0.01). Supplementary Figure S5: Cebpa has increased expression in expanding iWAT compared to gWAT. Taqman qPCR for Cebpa in RNA extracted from whole iWAT or gWAT (n = 5–7) in Cebpa_fl/fl mice at 10 weeks of age (chow‐fed), after 20 weeks of additional chow feeding, or after 20 weeks of additional HFD feeding. Student's t‐test was used to analyze results (*p < 0.05, **p < 0.01). Supplementary Figure S6: WAT mass in Cebpa_ASKO mice reduces over time regardless of diet. Masses of white adipose depots expressed as percentage of body weight (n = 7–11) in male Cebpa_ASKO mice at 10 weeks of age (chow‐fed), after 20 weeks of additional chow feeding, or after 20 weeks of additional HFD feeding. Student's t‐test was used to analyze results (*p < 0. [file OBY-34-882-s001.docx]

**Supplemental Information**

**C/EBPα Is Essential for Gonadal but Not Inguinal White Adipose Tissue Formation in Mice**

Krista Y. Hu^1^, Yu-Lin Ma^1^, Esme A. Dodge^1^, Olivia A. B. Maguire^1^, Caio V. Matias^1^, Ryan P. Barney^2^, Hector S. Himede^1^, Juliana Gomez Pardo^1^, Miriam Cepeda^1^, Scott M. Gordon^2,3^, Robert C. Bauer^1^*

^1^Cardiometabolic Genomics Program, Division of Cardiology, Department of Medicine, Columbia University, New York, NY 10032

^2^Department of Physiology, University of Kentucky, Lexington, KY

^3^Saha Cardiovascular Research Center, University of Kentucky, Lexington, KY

**Supplemental Figures**

**
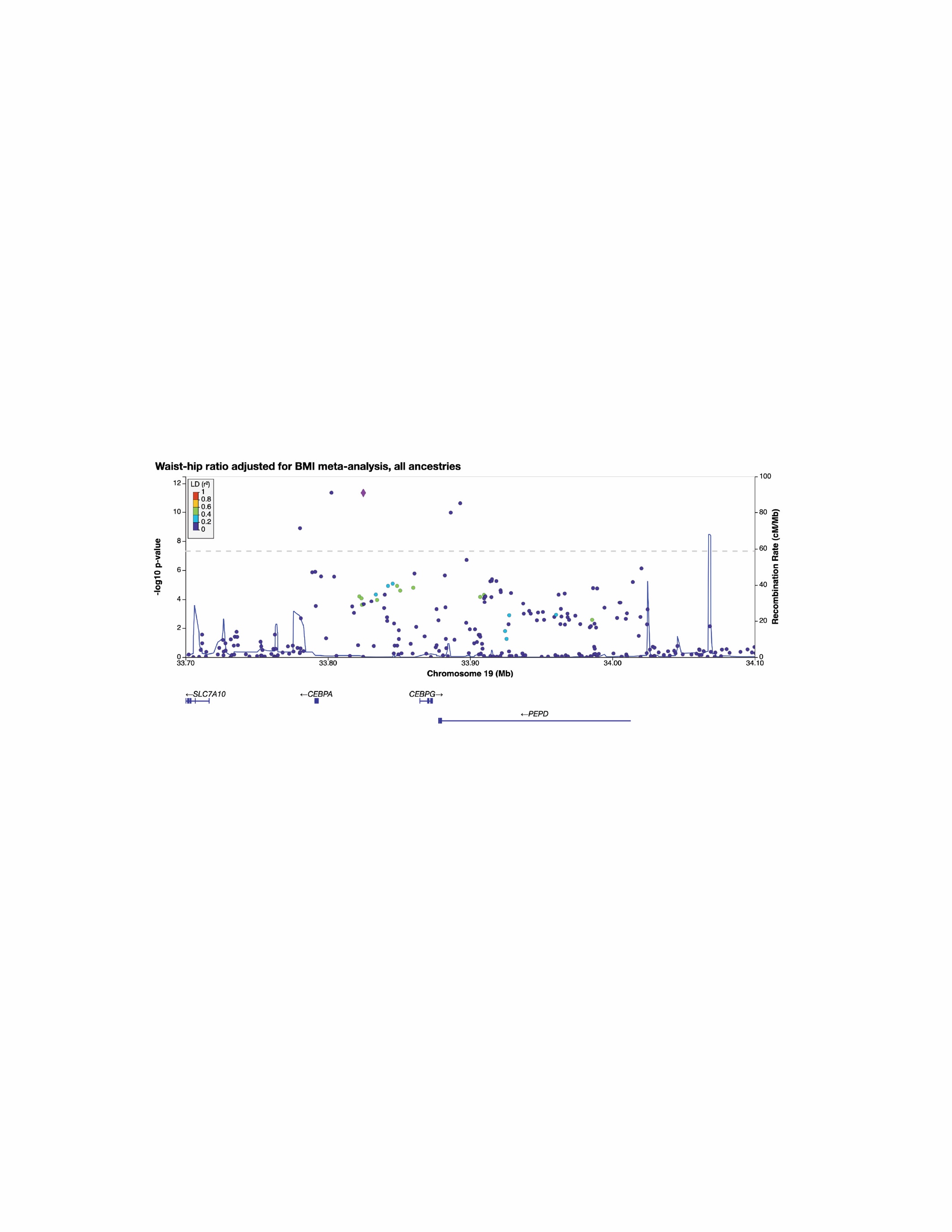
**

**Supplementary Figure S1: The *CEBPA* GWAS locus.** LocusZoom plot of SNPs at the 19q13 locus associated with waist-to-hip ratio adjusted for BMI from the GIANT consortium [55].

**
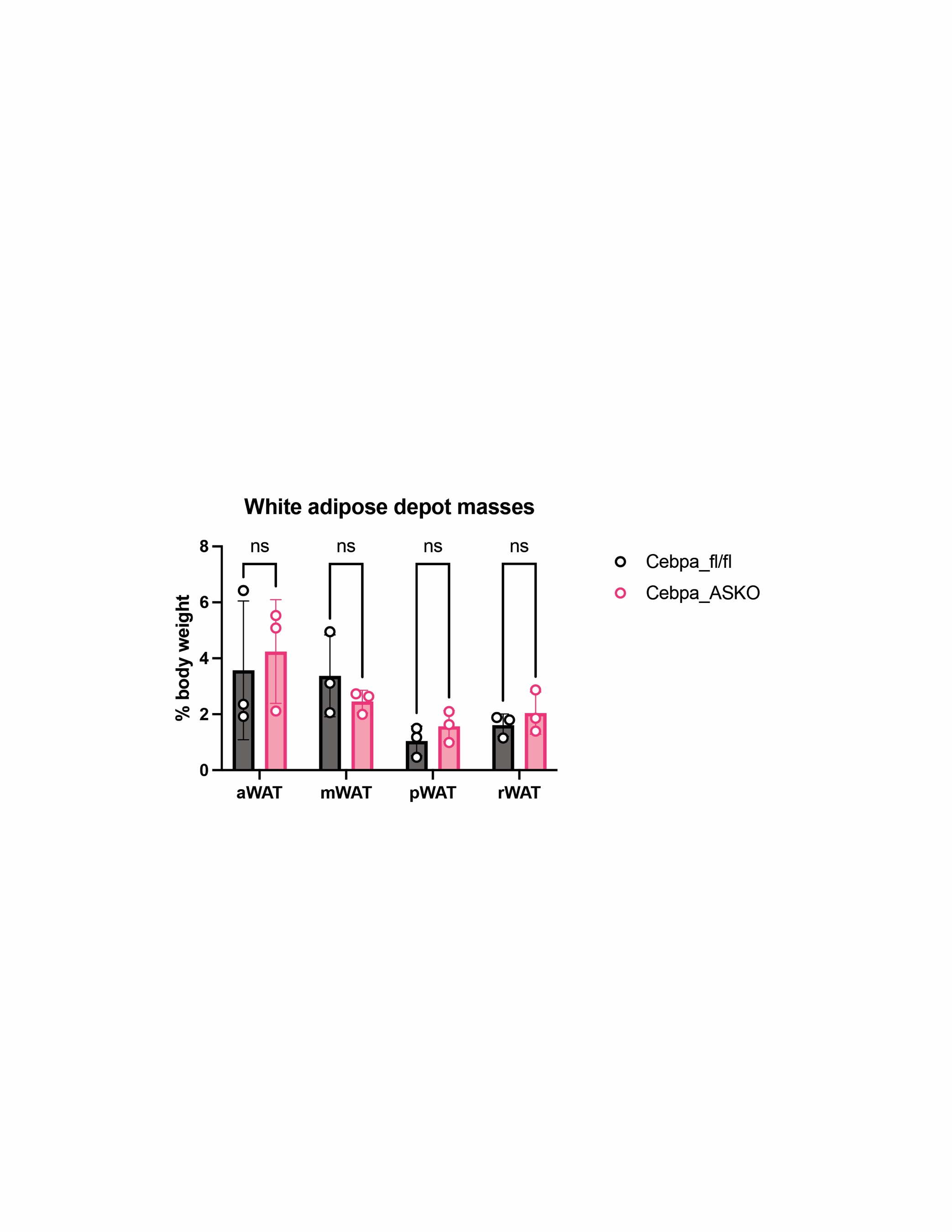
**

**Supplementary Figure S2: Cebpa_ASKO mice have no change in other WAT depot masses. A.** Axillary, mesenteric, perirenal, and retroperitoneal adipose depot masses as percent body weight in male mice at 10-12 weeks of age (n=3). All mice were chow-fed. Student’s t-test was used to analyze results.

**
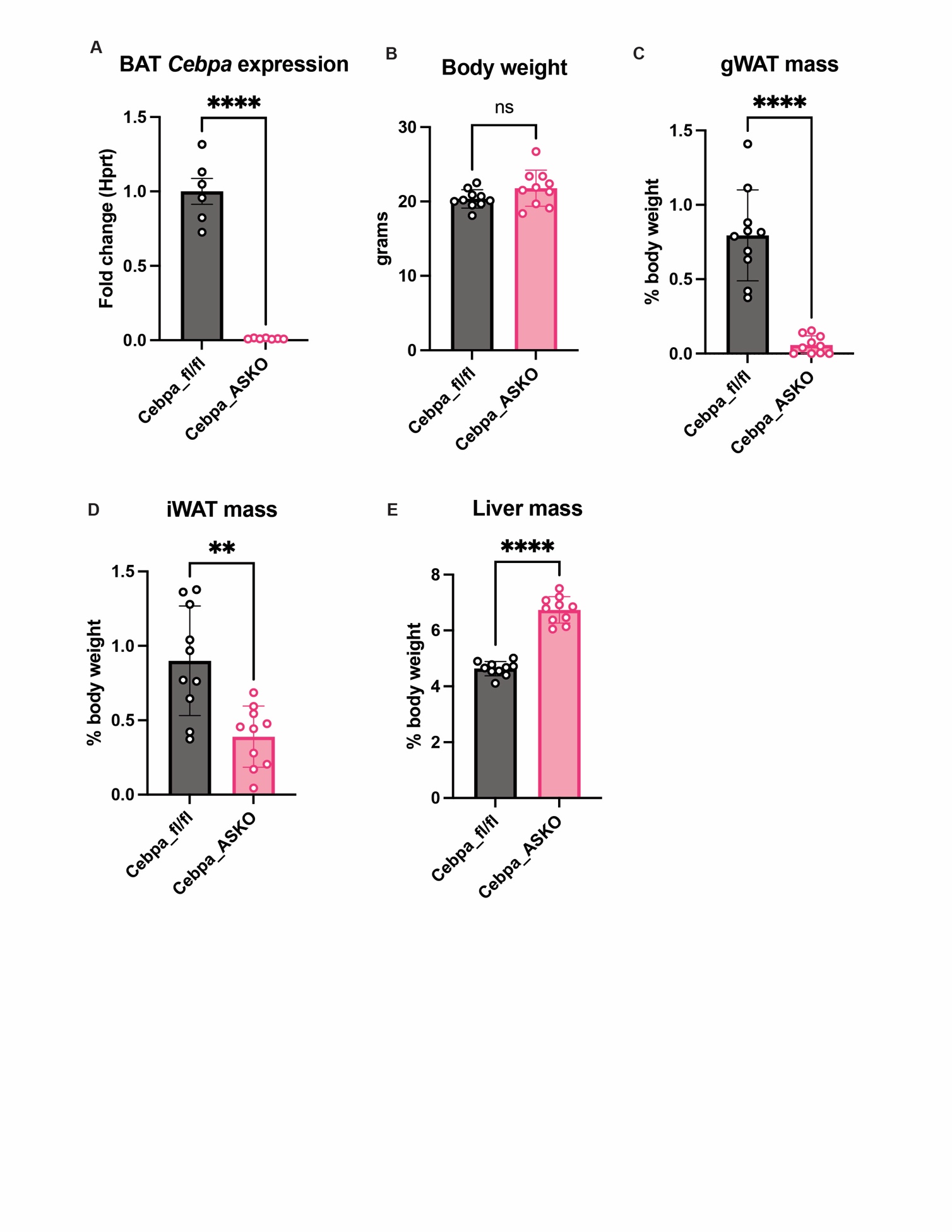
**

**Supplementary Figure S3: Female Cebpa_ASKO mice have reduced gWAT mass. A.** TaqMan qPCR of *Cebpa* expression in (iwat) and BAT from 10-12-week-old female Cebpa_fl/fl and Cebpa_ASKO mice (n=6-7). **B.** Body weights at 10-12 weeks of female Cebpa_fl/fl and Cebpa_ASKO mice (n=10). **C-E.** gWAT, iWAT, and liver mass as percent body weight at 10-12 weeks of age (n=10). All mice were chow-fed. Student’s t-test was used to analyze results (**p<0.01, ****p<0.0001).

**
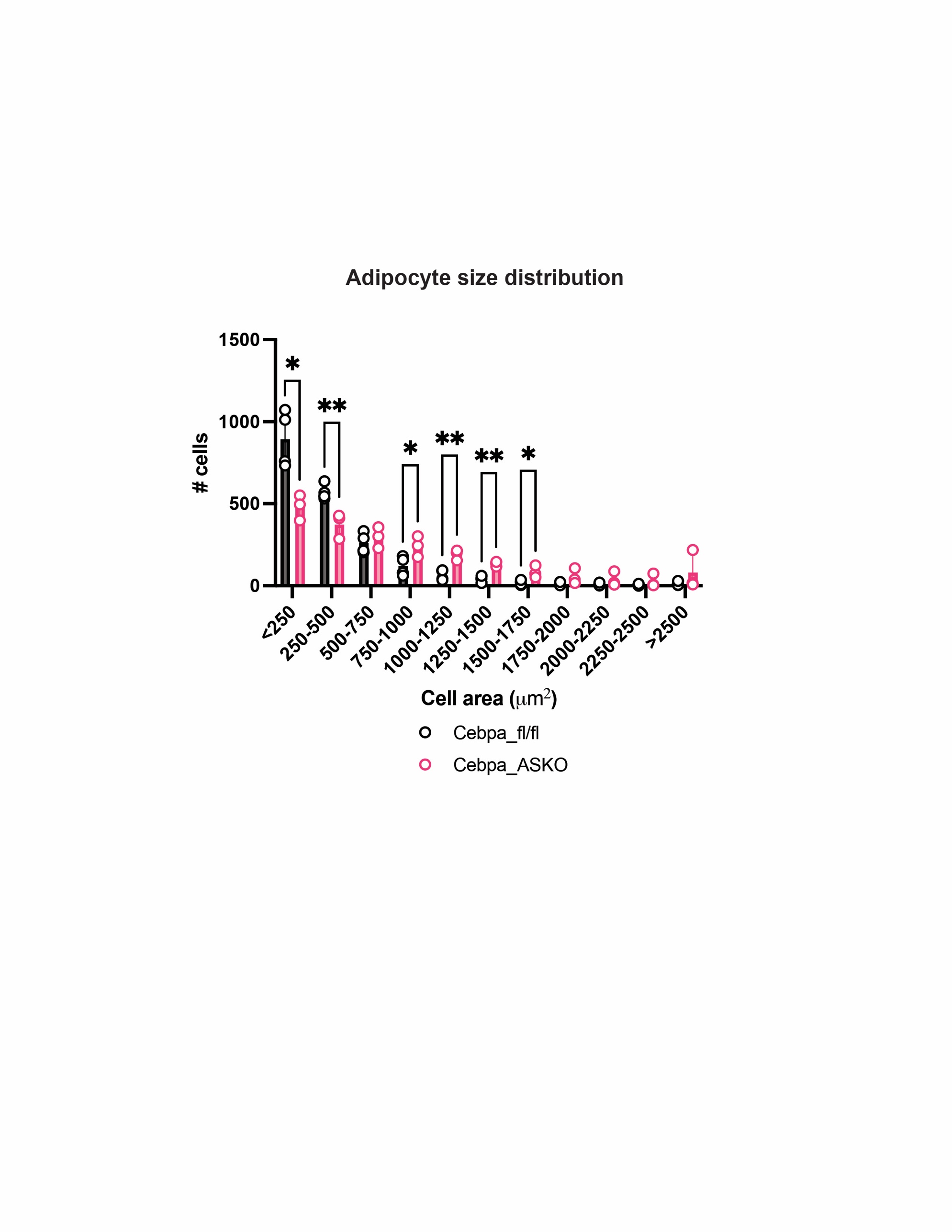
**

**Supplementary Figure S4: Cebpa_ASKO mice have larger iWAT adipocytes. A**. Size distribution of adipocytes calculated using the Adiposoft FIJI plugin from representative iWAT H&E images of 10–12-week-old mice (n=3). All mice were chow-fed. Student’s t-test was used to analyze results (*p<0.05, **p<0.01).

**
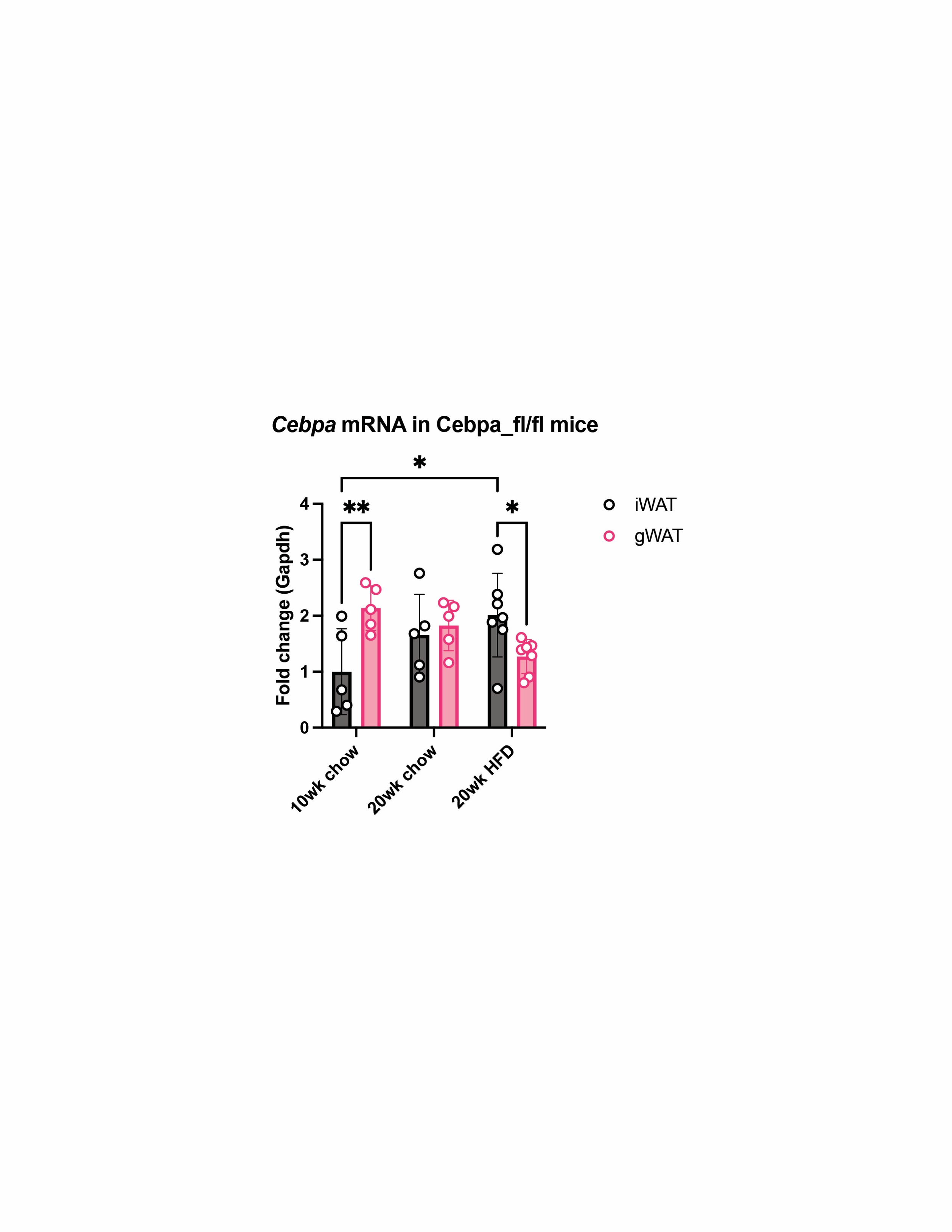
**

**Supplementary Figure S5: Cebpa has increased expression in expanding iWAT compared to gWAT.** Taqman qPCR for Cebpa in RNA extracted from whole iWAT or gWAT (n=5-7) in Cebpa_fl/fl mice at 10wks of age (chow-fed), after 20 weeks of additional chow feeding, or after 20 weeks of additional HFD feeding. Student’s t-test was used to analyze results (*p<0.05, **p<0.01).

**
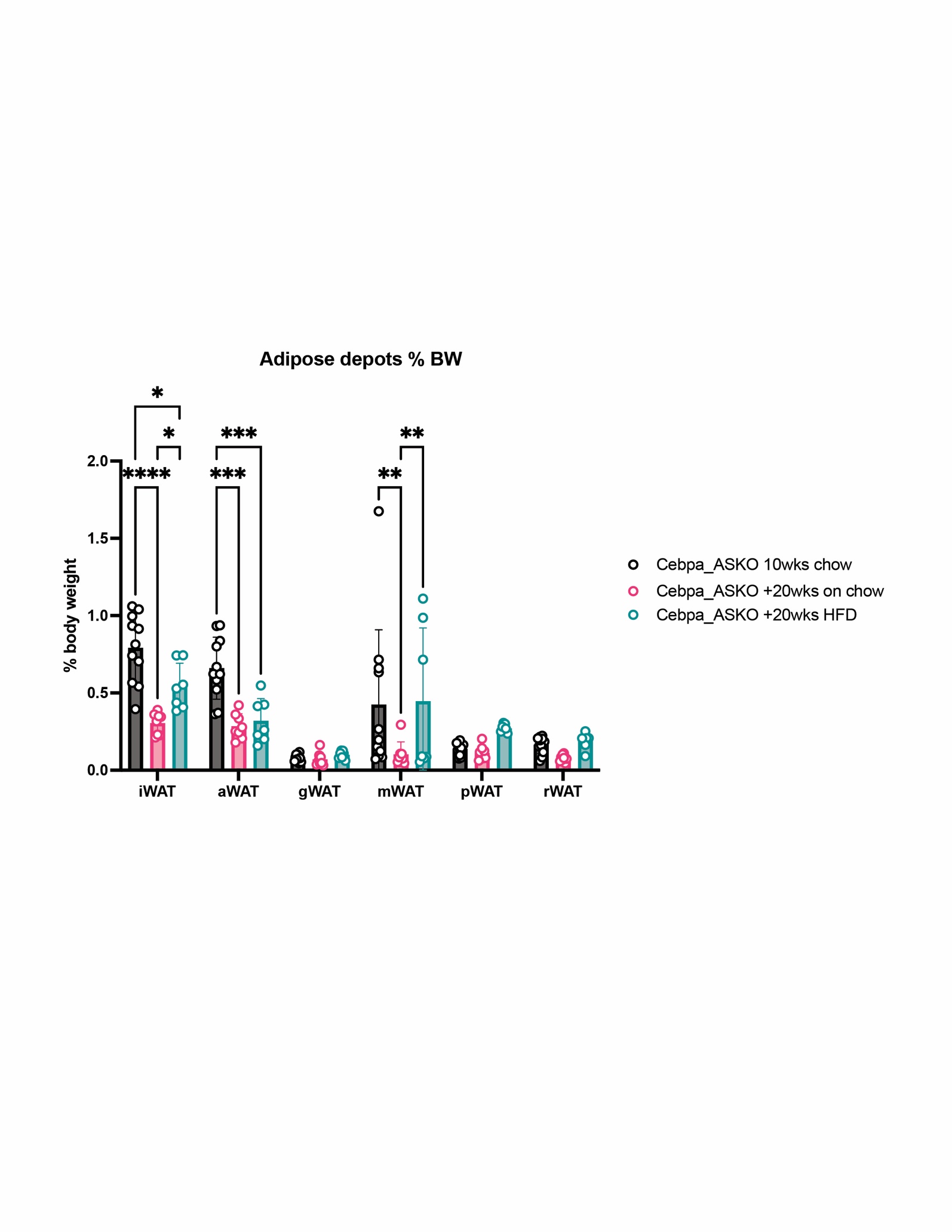
**

**Supplementary Figure S6: WAT mass in Cebpa_ASKO mice reduces over time regardless of diet.** Masses of white adipose depots expressed as percentage of body weight (n=7-11) in male Cebpa_ASKO mice at 10wks of age (chow-fed), after 20 weeks of additional chow feeding, or after 20 weeks of additional HFD feeding. Student’s t-test was used to analyze results (*p<0.05, **p<0.01, ***p<0.001).

**
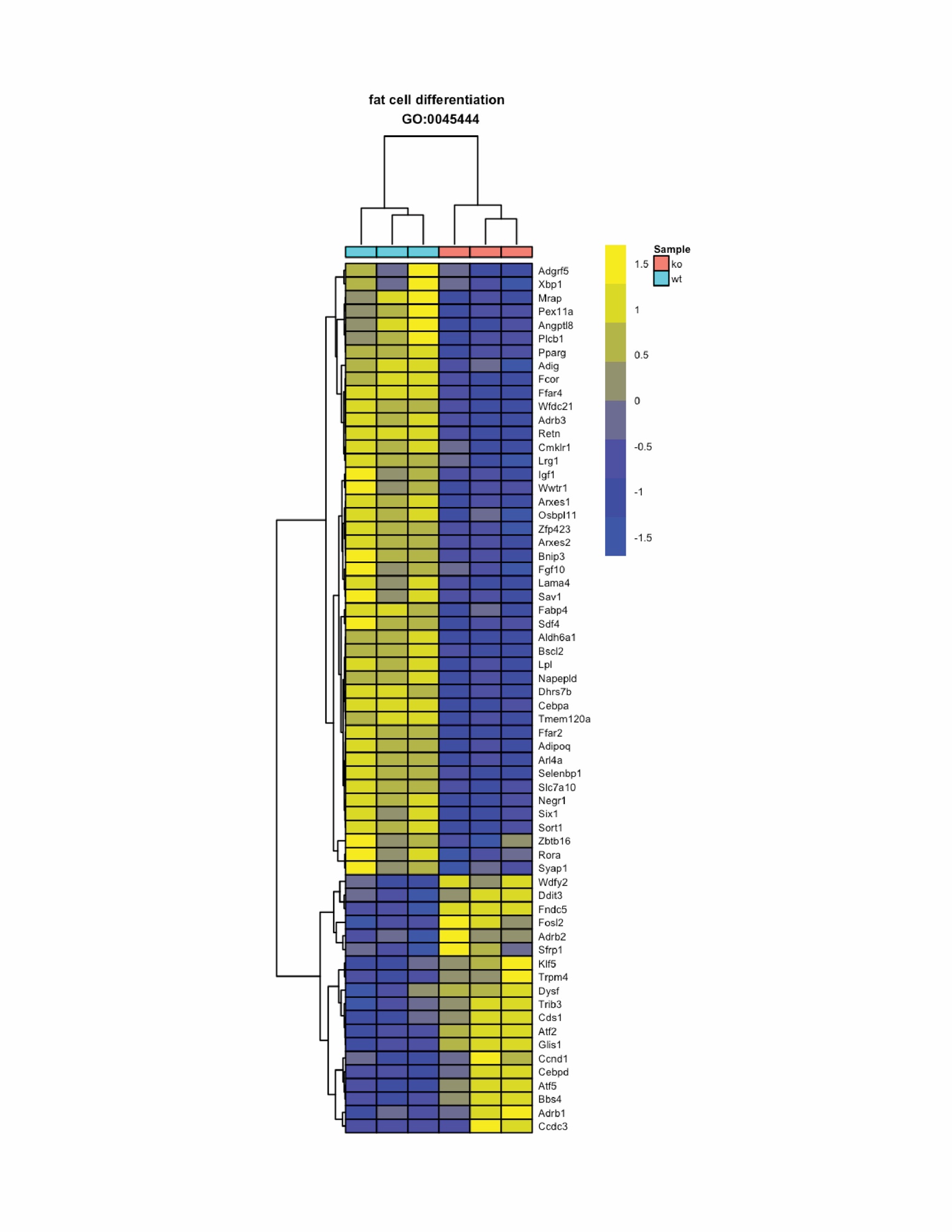
**

**Supplementary Figure S7: Cebpa_ASKO iWAT has changes in expression of adipocyte differentiation genes.** Heatmap of expression of genes clustered in the fat cell differentiation GO term from bulk RNA-seq data (n=3). All mice were chow-fed.

**
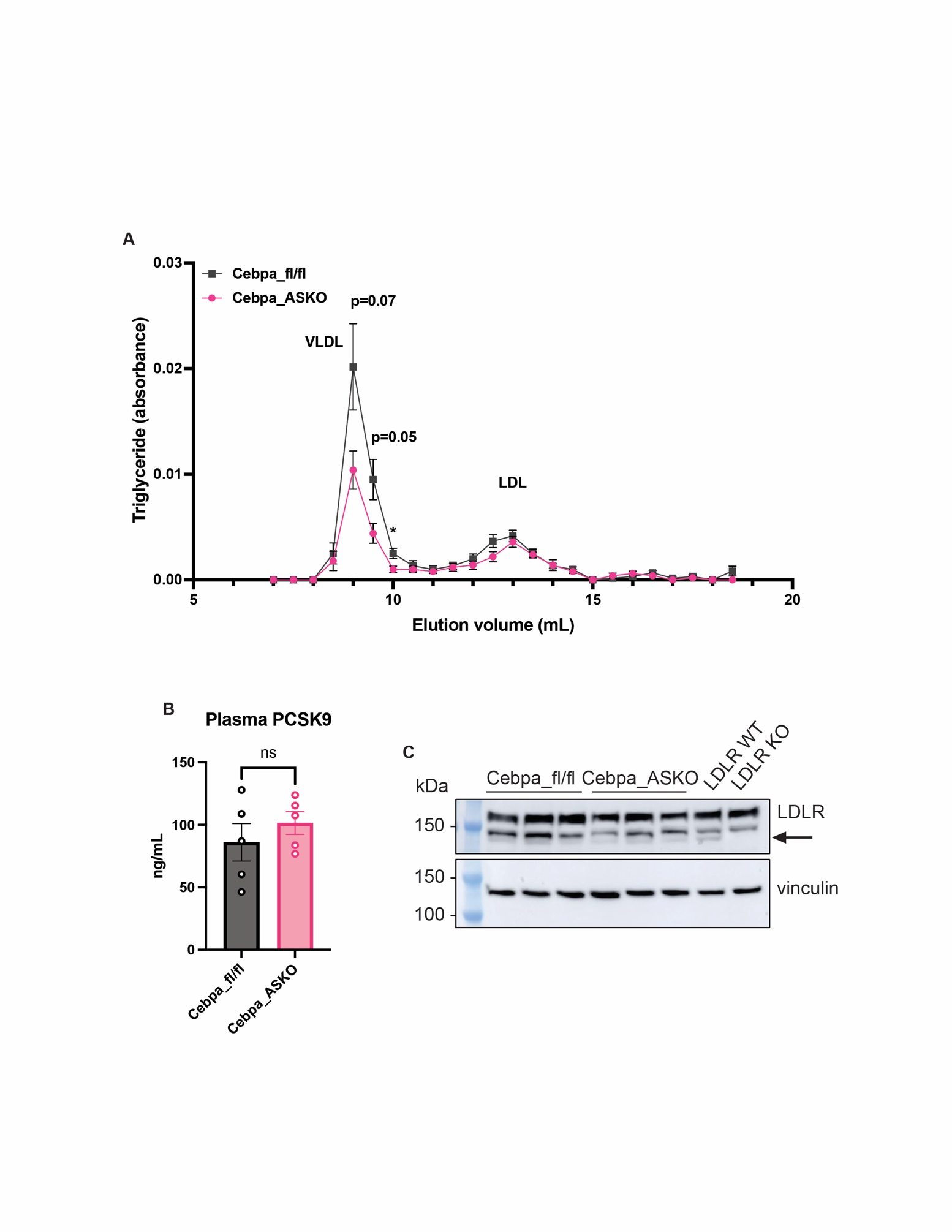
**

**Supplementary Figure S8: Cebpa_ASKO mice exhibit normal hepatic LDLR pathway function.** A: Triglyceride (TG) FPLC profile of pooled plasma (n=5-6) from 4h fasted male Cebpa_fl/fl and Cebpa_ASKO mice. B: Plasma PCSK9 levels measured via ELISA in 4h fasted mice (n=5). C**:** Western blot analysis of hepatic LDLR protein levels. Student’s t-test was used to analyze results (****p<0.0001).

**
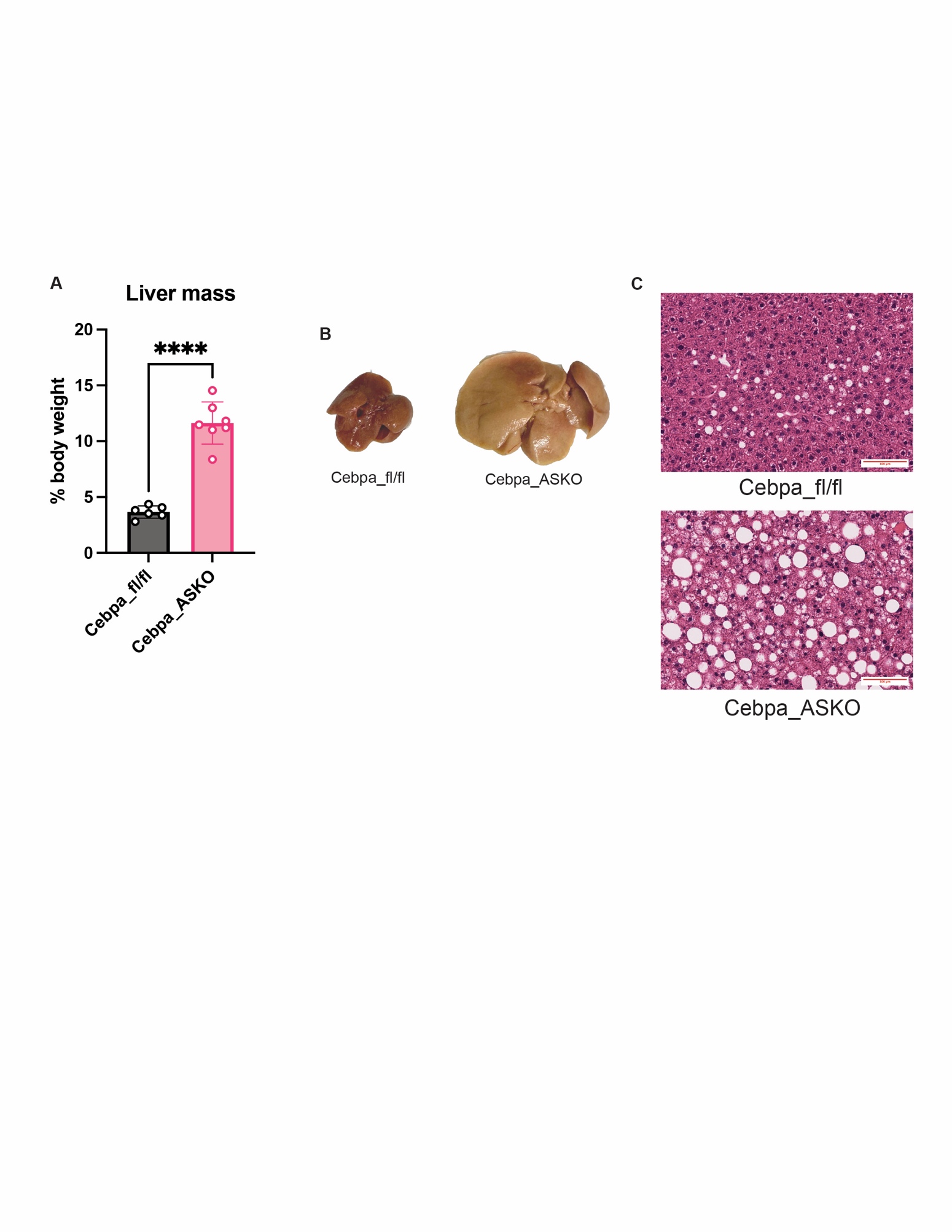
**

**Supplementary Figure S9: High fat diet feeding exacerbates the lipid deposition in livers of Cebpa_ASKO mice. A**. Liver mass measured in HFD-fed Cebpa_fl/fl and Cebpa_ASKO mice. **B.** Macroscopic image of livers from HFD-fed Cebpa_fl/fl and Cebpa_ASKO mice. **C.** Representative images from H&E-stained livers. All mice were fed with HFD for 20 weeks. Student’s t-test was used to analyze results (****p<0.0001).

**
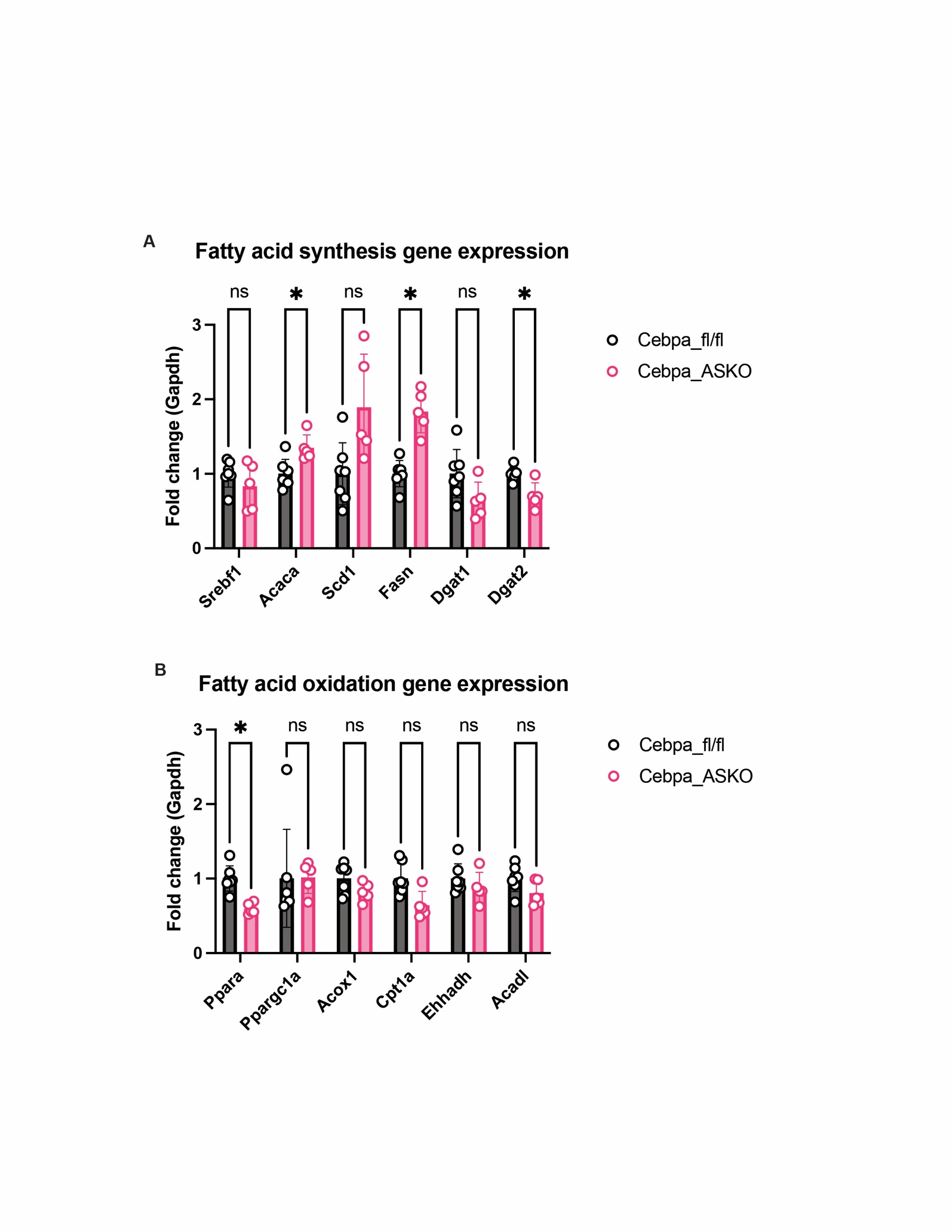
**

**Supplementary Figure S10: Cebpa_ASKO mice have increased expression of hepatic triglyceride synthesis genes.** A. Taqman qPCR for Srebf1, Acaca, Scd1, Fasn, Dgat1, and Dgat2 in RNA extracted from whole livers of chow-fed male Cebpa_fl/fl and Cebpa_ASKO mice at 10wks of age (n=5-6). B. Taqman qPCR for Ppar, Ppargc1a, Acox1, Cpt1a, Ehhadh, and Acadl in RNA extracted from whole livers of chow-fed male Cebpa_fl/fl and Cebpa_ASKO mice at 10wks of age (n=5-6). Student’s t-test was used to analyze results (*p<0.05).

**Supplemental Methods:**

*SVF isolation and culture*

Inguinal white adipose tissue (iWAT) was harvested from 3-5 8-10-week-old sex and age-matched mice and placed in ice-cold 1X PBS. Lymph nodes were removed. iWAT was pooled per genotype and minced manually for 5 minutes in digestion media (L-15 Leibovitz media, 1.5% BSA, 1% Pen/Strep, 10 U/mL DNAseI, 480 U/mL hyaluronidase, 0.14 U/mL Liberase TM). Tissue was dissociated by shaking in digestion buffer at 37C for 1 hour at 250 rpm. Homogenate was filtered through a 100 uM cell strainer and spun at 300 x g, 4C, for 10 mins. Supernatant was discarded, and the cell pellet was washed in 10mL cell culture medium (DMEM, 10% FBS, 1% Pen/Strep, 2mM L-Glut), then resuspended in 5mL cell culture medium supplemented with 1 ug/mL insulin. Cell resuspension was plated in a 10cm collagen-coated dish and allowed to grow until 95% confluence, with media replaced every 2-3 days. Then, cells were lifted with 0.25% trypsin, counted, and seeded into either 6-well or 12-well plates. Once cells reached 95% confluence, differentiation was initiated with a cocktail consisting of 10% FBS, 1% Pen/Strep, 5 ug/mL insulin, 1 uM rosiglitazone, 1 uM dexamethasone, and 250 uM IBMX in DMEM/F12. After 48 hours in differentiation cocktail, media was changed to maintenance media consisting of 10% FBS, 1% Pen/Strep, 5 ug/mL insulin, and 1 uM rosiglitazone in DMEM/F12. Experiments were started after day 7 of differentiation unless otherwise noted.

*Oil red O staining*

Oil red O (ORO) staining was performed on cell cultures in 12-well plates. Cells were washed twice with 2X PBS before fixing in 10% paraformaldehyde for 30mins at room temperature. Cells were washed twice for with water for 1 min each, then once with 60% isopropanol for 5 mins. Cells were stained with 60% ORO stock solution in water for 15 mins, then washed 3 times with water for 2 mins each before imaging. To quantify ORO area, water was aspirated, and the cells were incubated with 100% isopropanol for 3 mins to dislodge neutral lipids into solution. The isopropanol was then collected and added in equal volumes to a 96-well plate before measuring absorbance at 510nm.

*Fast protein liquid chromatography analysis*

Plasma (50 μL/mouse) was loaded onto a single Superose 6 Increase (10/300 GL) column using an Akta Pure 25L instrument. Samples were eluted through the column at 0.75 mL/min in phosphate buffered saline and collected as 0.5 mL fractions in 96-well plates. Fractions were analyzed for total cholesterol and triglyceride content using colorimetric enzymatic assays. Data are reported as mean absorbance +/- SEM.

*Bulk RNA-seq of inguinal scWAT and livers*

10-week-old female mice were euthanized and 50-80mg of inguinal scWAT was dissected out and placed into ice-cold PBS. Livers were dissected from 10-week-old male mice. Tissues were homogenized in Qiazol Lysis Reagent, and RNA was isolated using the RNeasy Lipid Tissue Mini Kit (Qiagen). Quality was assessed with TapeStation before submission to the Columbia Genome Center for bulk, paired-end RNA-sequencing (NextSeq 500) and differential gene expression analysis (DESeq2). RNA-seq data is available from GEO (GSE302944).

*Histology*

For adipose tissue and liver histology, whole tissues or lobes from Cebpa_fl/fl and Cebpa_ASKO mice and neonates were fixed in 4% PFA for 24 hours before switching to 70% ethanol. Tissues were submitted to the Columbia Molecular Pathology Shared Resource Histology Service, where they were embedded flat in paraffin and sectioned at 7um (neonate tissue) or 8um (adult tissue), for 3 serial sections per slide, followed by H&E staining. The histology service provided whole slide scanning at 40X (Leica SCN 400). Adipose tissue images were analyzed using the Adiposoft ImageJ plugin (parameters: minimum diameter = 10um, maximum diameter = 100um). Adipocyte number calculations were done using the Goldrick formula.

*Quantitative PCR*

RNA from adipose and liver tissues was isolated using the RNeasy Lipid Tissue Mini kit (Qiagen). cDNA was synthesized with the High-Capacity cDNA Reverse Transcription Kit (Applied Biosystems). qPCR was performed with pre-designed TaqMan probes from Thermo Fisher Scientific (see below) and TaqMan Fast Advanced Master Mix (Applied Biosystems).

Pre-designed Taqman probes for qPCR

| **Probe** | **Manufacturer** | **Assay ID** |
| --- | --- | --- |
| Taqman *Acaca* probe | Thermo Fisher Scientific | Mm01304257_m1 |
| Taqman *Acadl* probe | Thermo Fisher Scientific | Mm01256456_m1 |
| Taqman *Acox1* probe | Thermo Fisher Scientific | Mm01246834_m1 |
| Taqman *Adipoq* probe | Thermo Fisher Scientific | Mm00456425_m1 |
| Taqman *Cebpa* probe | Thermo Fisher Scientific | Mm00514283_s1 |
| Taqman *Cebpb* probe | Thermo Fisher Scientific | Mm00843434_s1 |
| Taqman *Cidea* probe | Thermo Fisher Scientific | Mm00432554_m1 |
| Taqman *Cpt1a* probe | Thermo Fisher Scientific | Mm01231183_m1 |
| Taqman *Dgat1* probe | Thermo Fisher Scientific | Mm00515643_m1 |
| Taqman Dgat2 probe | Thermo Fisher Scientific | Mm00499536_m1 |
| Taqman *Ehhadh* probe | Thermo Fisher Scientific | Mm00619685_m1 |
| Taqman *Fasn* probe | Thermo Fisher Scientific | Mm006662319_m1 |
| Taqman *Gapdh* probe | Thermo Fisher Scientific | Mm99999915_g1 |
| Taqman *Hprt* probe | Thermo Fisher Scientific | Mm00446968_m1 |
| Taqman *Lep* probe | Thermo Fisher Scientific | Mm00434759_m1 |
| Taqman *Pparg* probe | Thermo Fisher Scientific | Mm00440940_m1 |
| Taqman *Ppargc1* probe | Thermo Fisher Scientific | Mm01208835_m1 |
| Taqman *Prdm16* probe | Thermo Fisher Scientific | Mm00712556_m1 |
| Taqman *Scd1* probe | Thermo Fisher Scientific | Mm00772290_m1 |
| Taqman *Srebf1* probe | Thermo Fisher Scientific | Mm00550338_m1 |
| Taqman *Ucp1* probe | Thermo Fisher Scientific | Mm01244861_m1 |

*Western blot*

Cells or tissues were homogenized in RIPA buffer supplemented with 1X Halt Protease and Phosphatase Inhibitor (Thermo Fisher Scientific 78440). Homogenates were clarified by centrifugation at 12,000 x g at 4C for 15mins twice. Protein in clarified homogenates was quantified by BCA assay (Thermo Fisher Scientific), and 20-30ug of protein was run on a premade 10% Bis-Tris SDS-page gel and transferred to a nitrocellulose membrane. Membrane was blocked in 5% milk and incubated in primary antibody overnight (see Supplemental Methods). Proteins were detected with HRP-linked secondary antibody and visualized through incubation in Immobilon ECL Ultra Western HRP Substrate (Millipore Sigma WBULS0100) or Luminata Classico Western HRP Substrate (Millipore Sigma WBLUC0500). Membranes were incubated in Restore Western Blot Stripping Buffer (Fisher Scientific PI21059) for 15 min before reblocking and subsequent reprobing.

Antibodies

| **Target** | **Manufacturer** | **Catalog number** | **Dilution** |
| --- | --- | --- | --- |
| C/EBPa | Cell Signaling Technology | 8178S | 1:1000 |
| ACRP30 | R&D Systems | AF1119 | 1:1000 |
| PPARg | Cell Signaling Technology | 2443S | 1:1000 |
| Phospho-HSL (Ser563) | Cell Signaling Technology | 4139S | 1:1000 |
| HSL | Cell Signaling Technology | 4107T | 1:1000 |
| ATGL | Cell Signaling Technology | 2138S | 1:1000 |
| Vinculin | Sigma-Aldrich | V9131 | 1:1000 |
| LDLR | Abcam | AB30532 | 1:1000 |
| IgG (H+L) Goat anti-mouse, HRP | Invitrogen | PI31430 | 1:3000 |
| IgG (H+L) Goat anti-rabbit, HRP | Invitrogen | PI31460 | 1:3000 |
| IgG (H+L) Rabbit anti-goat, HRP | Invitrogen | PI31402 | 1:7000 |
